# Delineating the heterogeneity of matrix-directed differentiation toward soft and stiff tissue lineages via single-cell profiling

**DOI:** 10.1073/pnas.2016322118

**Published:** 2021-05-03

**Authors:** Shlomi Brielle, Danny Bavli, Alex Motzik, Yoav Kan-Tor, Xue Sun, Chen Kozulin, Batia Avni, Oren Ram, Amnon Buxboim

**Affiliations:** ^a^Alexander Grass Center for Bioengineering, Hebrew University of Jerusalem, 9190401 Jerusalem, Israel;; ^b^Department of Cell and Developmental Biology, Hebrew University of Jerusalem, 9190401 Jerusalem, Israel;; ^c^Rachel and Selim Benin School of Computer Science and Engineering, Hebrew University of Jerusalem, 9190401 Jerusalem, Israel;; ^d^Department of Biological Chemistry, Hebrew University of Jerusalem, 9190401 Jerusalem, Israel;; ^e^Department of Bone Marrow Transplantation, Hadassah Medical Center, 91120 Jerusalem, Israel

**Keywords:** mechanobiology, single-cell analysis, mesenchymal stem cells, cell heterogeneity, tropomyosin

## Abstract

Mesenchymal stromal/stem cells (MSCs) form a heterogeneous population of multipotent progenitors that contribute to tissue regeneration and homeostasis. MSCs assess extracellular elasticity by probing resistance to applied forces via adhesion, cytoskeletal, and nuclear mechanotransducers that direct differentiation toward soft or stiff tissue lineages. Even under controlled culture conditions, MSC differentiation exhibits substantial cell-to-cell variation that remains poorly characterized. By single-cell transcriptional profiling of nonconditioned, matrix-conditioned, and early differentiating cells, we identified distinct MSC subpopulations with distinct mechanosensitivities, differentiation capacities, and cell cycling. We show that soft matrices support adipogenesis of multipotent cells and early endochondral ossification of nonadipogenic cells, whereas intramembranous ossification and preosteoblast proliferation are directed by stiff matrices. Using diffusion pseudotime mapping, we outline hierarchical matrix-directed differentiation and perform whole-genome screening of mechanoresponsive genes. Specifically, top-ranked tropomyosin-1 is highly sensitive to stiffness cues both at RNA and protein levels, and changes in TPM1 expression determine the differentiation toward soft versus stiff tissue lineage. Consistent with actin stress fiber stabilization, tropomyosin-1 overexpression maintains YAP1 nuclear localization, activates YAP1 target genes, and directs osteogenic differentiation. Knockdown of tropomyosin-1 reversed YAP1 nuclear localization consistent with relaxation of cellular contractility, suppressed osteogenesis, activated early endochondral ossification genes after 3 d of culture in induction medium, and facilitated adipogenic differentiation after 1 wk. Our results delineate cell-to-cell variation of matrix-directed MSC differentiation and highlight tropomyosin-mediated matrix sensing.

## Significance

The clinical utility of mesenchymal stromal/stem cells (MSCs) in mediating immunosuppressive effects and supporting regenerative processes is broadly established. However, the inherent heterogeneity of MSCs compromises its biomedical efficacy and reproducibility. To study how cellular variation affects fate decision-making processes, we perform single-cell RNA sequencing at multiple time points during bipotential matrix-directed differentiation toward soft- and stiff tissue lineages. In this manner, we identify distinctive MSC subpopulations that are characterized by their multipotent differentiation capacity and mechanosensitivity. Also, whole-genome screening highlights TPM1 as a potent mechanotransducer of matrix signals and regulator of cell differentiation. Thus, by introducing single-cell methodologies into mechanobiology, we delineate the complexity of adult stem cell responses to extracellular cues in tissue regeneration and immunomodulation.

Mesenchymal stromal/stem cells (MSCs) are present in all vascularized compartments owing to their perivascular origin, and as such they can be isolated from bone marrow, fat, placenta, and other tissues (1, 2). The definition of MSCs relies on their surface adherence and expansion in culture, the expression of several mesodermal and absence of hematopoietic surface markers, and retaining a multilineage differentiation capacity toward fat, cartilage, and bone under defined induction media. This elusive definition permits a significant molecular and phenotypic variation between cells that were derived from different donors, different tissues of the same donor, different clones isolated from the same tissue, and different cells of the same clone (3–5). MSCs have drawn considerable clinical interest for mediating immunomodulatory effects and for their “stemness.” (6) However, the inconsistent clinical outcomes of MSC-based treatments may suggest that the therapeutic efficacy of MSCs is compromised by their heterogeneous immunomodulatory and differentiation potential (7, 8). Characterizing MSC heterogeneity and its clinical implications will thus improve experimental reproducibility and biomedical standardization.

MSCs are highly sensitive to the mechanical properties of their microenvironment. These extracellular, tissue-specific cues are actively probed by all adherent cells (9–12), whereas impaired mechanosensitivity is leveraged by oncogenically transformed cells for evading apoptotic pathways (13). The mechanical resistance of the cellular microenvironment to cell-generated forces is set by extracellular elasticity and geometrical boundary conditions (14). These stress–strain relationships can be converted into biochemical signals through the forced unfolding of linker proteins (15, 16), force-sensitive (17) and catch-bond adhesions to extracellular matrix (ECM) ligands (18, 19) and to neighboring cell receptors (20, 21), tension-mediated filament stabilization (22, 23), or direct physical stretching of chromatin loci in the nucleus (24). The emerging intracellular signals are mediated via a number of pathways that regulate gene expression and direct cell fate decisions (25, 26). The resulting upregulation of cytoskeletal and force-generating target genes stabilizes a contractile cell state with positive feedback to extracellular stiffness (27, 28).

Here, we exposed bone marrow–derived MSCs, which had been harvested for bone marrow transplantation treatments (*Methods*), to matrices with controlled elasticities and to a bipotential induction mixture that permits differentiation toward fat or bone. To gain insight into the implications of MSC heterogeneity on cellular mechanosensitivity and multipotency, we transcriptionally profiled the cells via whole-genome single-cell RNA sequencing at nonconditioned, matrix-conditioned, and early differentiating stages. Unsupervised clustering of MSC subpopulations and diffusion pseudotime mapping revealed a bifurcation of cell state propagation between differentiated and nondifferentiated fates. Whole-genome screening highlighted tropomyosin-1 (TPM1) as a matrix-responsive gene, which was experimentally validated. Using targeted gene silencing and overexpression, TPM1 was found to be a highly potent regulator of cell differentiation downstream of tissue-level matrix mechanics. Characterizing cell-to-cell variations among the response to matrix and differentiation cues during cell state propagation contributes to elucidating MSC heterogeneity with future implications to cell-based therapeutics.

## Results

### Cell-To-Cell Variation in MSC Mechanosensitivity.

**Fig. 1.**
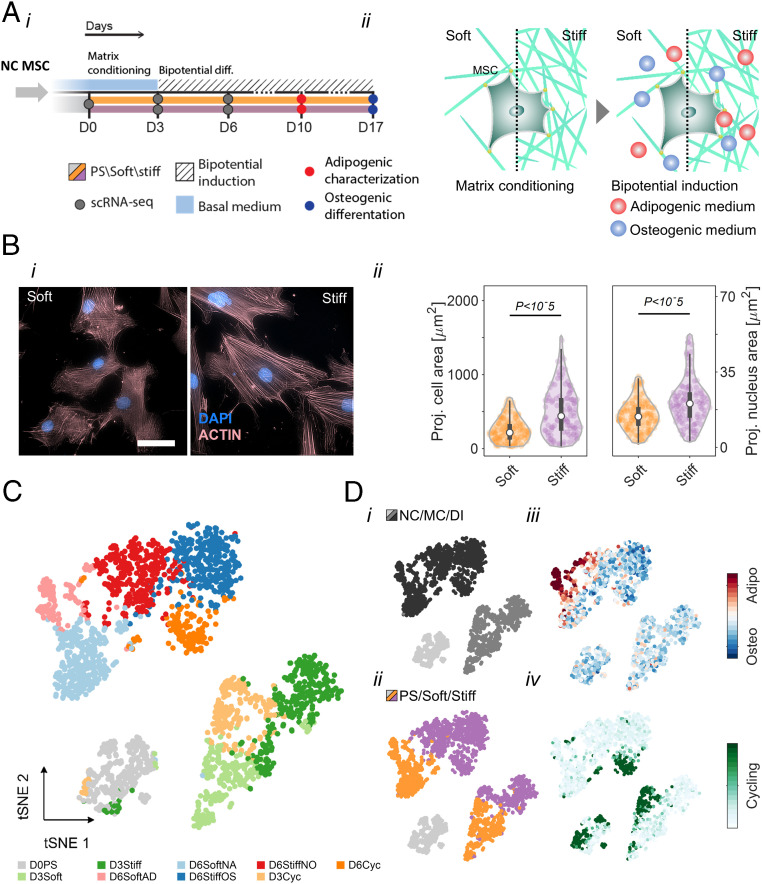
Resolving MSC heterogeneity using matrix elasticity and bipotential differentiation induction signals. (*A*, *i*) Experimental design: Nonconditioned (NC) MSCs were seeded on Day 0 (D0) on soft (2 kPa) and stiff (25 kPa) collagen-coated hydrogel matrices that mimic fat and that mimic the cartilaginous endochondral ossification/osteoid microenvironment, respectively. (*A*, *ii*) Following 3 d of matrix conditioning in basal medium, cells were cultured in adipo-osteogenic bipotential induction medium to study matrix-directed lineage commitment. Cell fate decisions toward adipogenic or osteogenic differentiation was evaluated on day 10 and day 17, respectively. Nonconditioned, matrix-conditioned, and early differentiating MSCs were harvested on day 0 (388 cells), day 3 (soft: 467 cells; stiff: 450 cells), and day 6 (soft: 534 cells; stiff: 951 cells), respectively and analyzed by single-cell transcriptional profiling. (*B*, *i*) Phalloidin staining of matrix-conditioned MSCs (male donor, age 40) exhibit striated organization of mature actomyosin stress fibers on stiff matrices and loosely organized F-actin networks on soft matrix. (Scale bar, 50 μm.) (*B*, *ii*) Distributions of cell and nucleus projected area are compared between soft and stiff matrices. (*C*) Single-cell transcriptomes of MSCs (male donor, age 40) were divided into nine subpopulations using unsupervised k-means clustering at the dimensionality reduced principal component space. Associations between subpopulations were projected onto a t-SNE map. (*D*) MSC subpopulations are characterized by (*i*) cell state, (*ii*) matrix elasticity, (*iii*) early differentiation, and (*iv*) cell cycling. NC: nonconditioned, MC: matrix conditioning, and DI: differentiation induction.

MSC differentiation toward soft and stiff tissue lineages is a tightly regulated process that integrates mechanical inputs and biochemical cues from the microenvironments (29–31). Here we studied low-passage MSCs that were obtained from bone marrow donors during allogeneic transplantation. To study how matrix elasticity directs differentiation toward adipogenesis or osteogenesis leading to fat and bone lineages, respectively, we expanded the MSCs on polystyrene and seeded them on collagen-coated hydrogel substrates with controlled stiffness: The “soft” collagen-coated hydrogel matrix (2 kPa) mimics the elasticity of adipose tissue (32), and the “stiff” chondrogenic pericellular matrix and osteoid matrix (25 kPa) (29, 33, 34) mimics the cartilaginous endochondral ossification/osteoid microenvironment (Fig. 1 *A*, *i*) (14). Cells were matrix conditioned in basal medium for 3 d before basal medium was replaced with bipotential induction medium that permits adipogenic and osteogenic differentiation (Fig. 1 *A*, *ii*) (35, 36). MSCs cultured on stiff matrix appear to spread more than those cultured on soft matrix (Fig. 1 *B*, *i*); the stiff matrix provides support for the striated organization of mature actomyosin stress fibers (37). Cell and nucleus projected areas are established markers of cell mechanosensitivity, yet only 25% of the cells spread more and nuclei became more stretched and flattened on stiff matrices than soft, thus reflecting cellular heterogeneity (Fig. 1 *B*, *ii*). Adipogenic differentiation was assessed via Nile-red staining of neutral lipid droplets in cells after 10 d of culture on soft and stiff matrices in different media (*SI Appendix*, Fig. S1 *A*, *i* and *ii*). Adipogenesis was favored on soft matrices under supportive medium conditions (*SI Appendix*, Fig. S1*B*). Similarly, osteogenic differentiation was evaluated via Alizarin-red staining of calcium deposition of cells after 17 d of culture on soft and stiff matrices in different media (*SI Appendix*, Fig. S1 *C*, *i*). During this period, the cells maintained a homogenous coverage and reached high confluence on all matrices (*SI Appendix*, Fig. S1 *C*, *ii*). Osteogenesis was quantified based on spectroscopic absorption of accumulated dye demonstrating the contributions of matrix stiffness in all medium conditions (*SI Appendix*, Fig. S1*D*). Despite the clear effects of matrix mechanics, most cells failed to undergo adipogenesis even under optimal adipogenic conditions, and a fraction of cells differentiated counter to matrix elasticity. Thus, population averages of matrix-directed cytoskeletal organization, cell and nucleus projected morphologies, and cell differentiation assays confirmed active mechanosensitivity (9), but the observed cell-to-cell variability is indicative of a heterogeneous response to mechanical cues as previously reported (4, 5, 38).

### Matrix Sensitivity, Early Differentiation, and Cell Cycling Define Distinctive MSC Subpopulations.

To characterize cell-to-cell variation in matrix-directed cell fate decisions, we employed microfluidics-based single-cell RNA sequencing (39, 40) and profiled transcriptomes of cells in the nonconditioned state (388 cells), matrix-conditioned state (soft: 467 cells, stiff: 450 cells), and early differentiation induction (soft: 534, stiff: 951 cells). Single-cell transcriptomes were dimensionally reduced via principal component analysis (PCA) of highly variable genes (*SI Appendix*, Fig. S2*A*). Transcriptomes clustered according to their cell state (nonconditioned, matrix-conditioned, and differentiation induction), and the corresponding technical replicates further clustered according to matrix elasticity (*SI Appendix*, Fig. S2*B*). All single-cell transcriptomes were divided into nine subpopulations using unsupervised k-means clustering in the PCA space and projected onto a t-distributed stochastic neighbor embedding (t-SNE) map (Fig. 1*C*). Matrix-conditioned cells analyzed on day 3 were divided between soft and stiff clusters (D3Soft and D3Stiff) and a cohort of cycling cells that were cultured on both matrices (D3Cyc). Early differentiation state cells, analyzed on day 6, were divided between adipogenic (D6SoftAD) and nonadipogenic (D6SoftNA) soft-matrix clusters and osteogenic (D6StiffOS) and nonosteogenic (D6StiffNO) stiff-matrix clusters. Unsupervised clustering paralleled the experimental parameters cell state and matrix elasticity (Fig. 1 *D*, *i* and *ii*) and correlated with established gene signatures of adipogenic (41, 42) and osteogenic (41, 43–46) differentiation and cell cycling (40) (Fig. 1 *D*, *iii* and *iv*). Unlike matrix-conditioned cycling subpopulation analyzed on day 3, the day 6 early differentiating cycling population consisted mainly of cells that were cultured on stiff matrix and enriched for osteogenic cells. Satisfyingly, the technical replicates of matrix-conditioned and early differentiating transcriptomes overlapped onto the t-SNE map in accordance with the specified subpopulations (*SI Appendix*, Fig. S2*C*). Cell state, matrix elasticity, differentiation, and cell cycling characterization of the clusters is summarized in Table 1.

**Table 1.** MSC subpopulations are defined by cell state, matrix elasticity, differentiation induction, and cell cycling

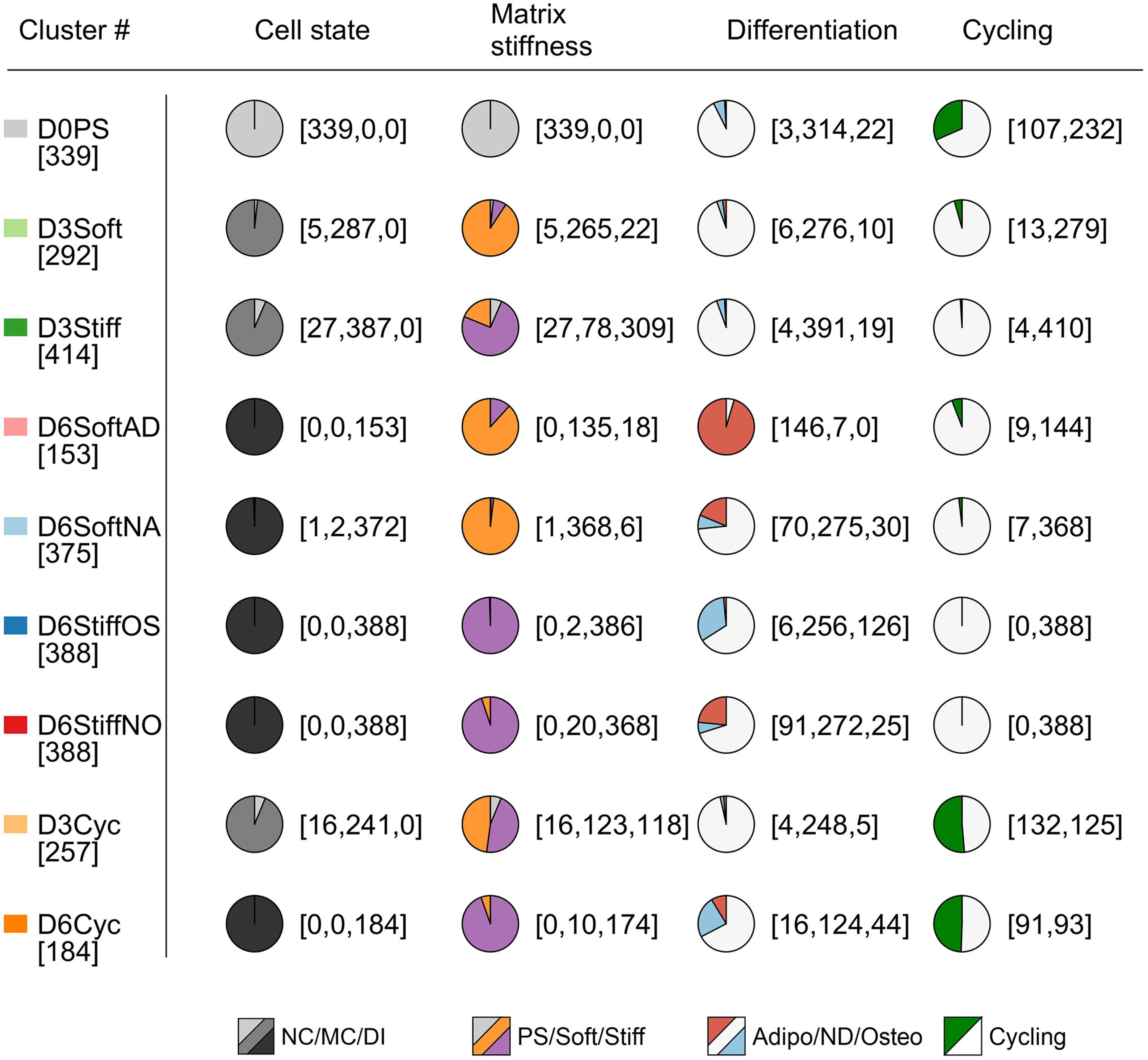

Subpopulations of single-cell transcriptomes are characterized by enrichment of day of culture, matrix elasticity, early differentiation, and cell cycling. Cell numbers are specified in brackets. PS: polystyrene, NC: nonconditioned, MC: matrix conditioning, DI: differentiation induction, Adipo: adipogenic, ND: nondifferentiated, Osteo: osteogenic.

**Fig. 2.**
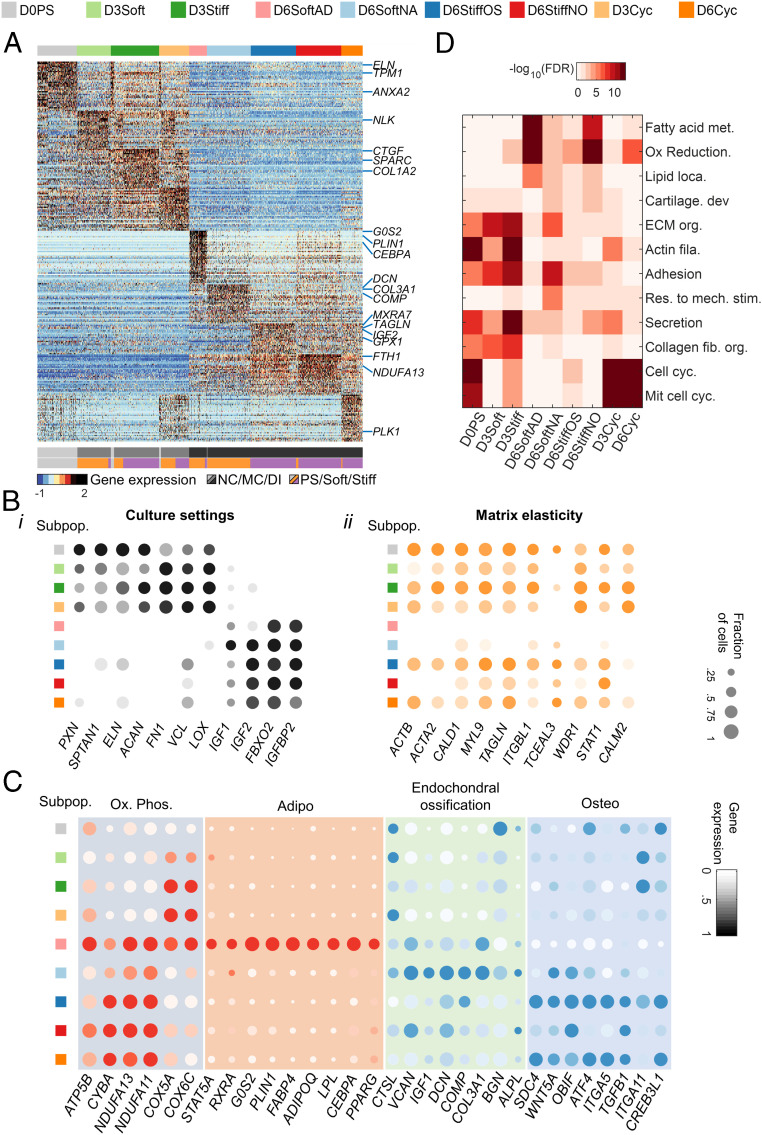
Differential gene expression analysis reveals cell fate decisions of MSC subpopulations. (*A*) Color-coded heatmap of *z*-score–normalized differentially expressed genes shows separation between nonconditioned, matrix-conditioned, and early differentiating subpopulations (lfc > 1; adjusted *P* value < 10^−5^). (*B*) Average intensities and fraction of positive cells are plotted for genes that are differentially expressed between (*i*) culture settings and (*ii*) matrix elasticities. (*C*) Day 6 subpopulations are characterized by expression patterns of OxPhos, adipogenesis, endochondral ossification, and osteogenesis. (*D*) GO term analysis of differentially expressed genes reveals enrichment of metabolic, cell adhesion, cytoskeletal, ECM, differentiation, and cell cycling patterns that characterize MSC subpopulations (Benjamini–Hochberg corrected). GO: Gene Ontology, ECM: extracellular matrix, and OxPhos: oxidative phosphorylation.

Clustering of gene intensities across single-cell transcriptomes highlights the associations of nonconditioned, matrix-conditioned, and early differentiating state subpopulations (Fig. 2*A*). Genes that were differentially expressed across cell states (Fig. 2 *B*, *i* and Dataset S1) underlie the transition from polystyrene to collagen-coated matrices (matrix conditioning) to bipotential induction medium (early differentiation). ECM and cell adhesion genes encoding paxillin, elastin, and aggrecan were highly expressed in nonconditioned cells, whereas genes encoding vinculin, fibronectin-1, and lysyl oxidase collagen cross-linker were upregulated in matrix-conditioned cells. Matrix adhesion and ECM genes were downregulated in early differentiating cells, and insulin growth factors 1 and 2 and the related binding protein gene *IGFBP2* were upregulated (Fig. 2 *B*, *i*). Next, we identified genes that were differentially expressed on soft versus stiff matrices (Fig. 2 *B*, *ii*). Stiff matrices supported upregulation of genes involved in actin binding and the actomyosin cytoskeleton during matrix conditioning in basal medium (D3Stiff versus D3Soft) and in nonosteogenic cells (D6StiffNO versus D6SoftAD) and nonadipogenic cells (D6StiffOS versus D6SoftNA) under bipotential induction conditions.

On soft matrix, upregulation of oxidative phosphorylation genes, which are associated with biogenesis during preadipocyte differentiation (47), parallels upregulation of adipogenic markers in D6SoftAD cells that differentiate into adipocytes (Fig. 2*C*). In nonadipogenic cells that were cultured also on soft matrices (D6SoftNA), nuclear-encoded oxidative phosphorylation genes are downregulated, and endochondral ossification markers are upregulated (Fig. 2*C*) (48). Endochondral ossification is linked with a low–oxidative phosphorylation state already during day 3 soft matrix conditioning (*SI Appendix*, Fig. S3 *A* and *B*). Unlike soft matrices that support adipogenesis and endochondral ossification, direct osteoblast differentiation via membranous ossification (D6StiffOS and D6Cyc cells) is supported by stiff matrices as indicated by upregulation of osteogenic markers. Gene ontology (GO) analysis showed enrichment of genes annotated with fatty acid metabolism, oxidative reduction, and lipid localization terms in D6SoftAD cells, in cartilage organization in D6SoftNA cells but not D6StiffOS cells, and in ECM, adhesion, and actin cytoskeletal terms in stiff-matrix– (D3Stiff) but not soft-matrix–conditioned cells (D3Soft) (Fig. 2*D*).

### Matrix-Directed Cell Fate Decision-Making Processes Revealed by Diffusion Pseudotime Mapping.

**Fig. 3.**
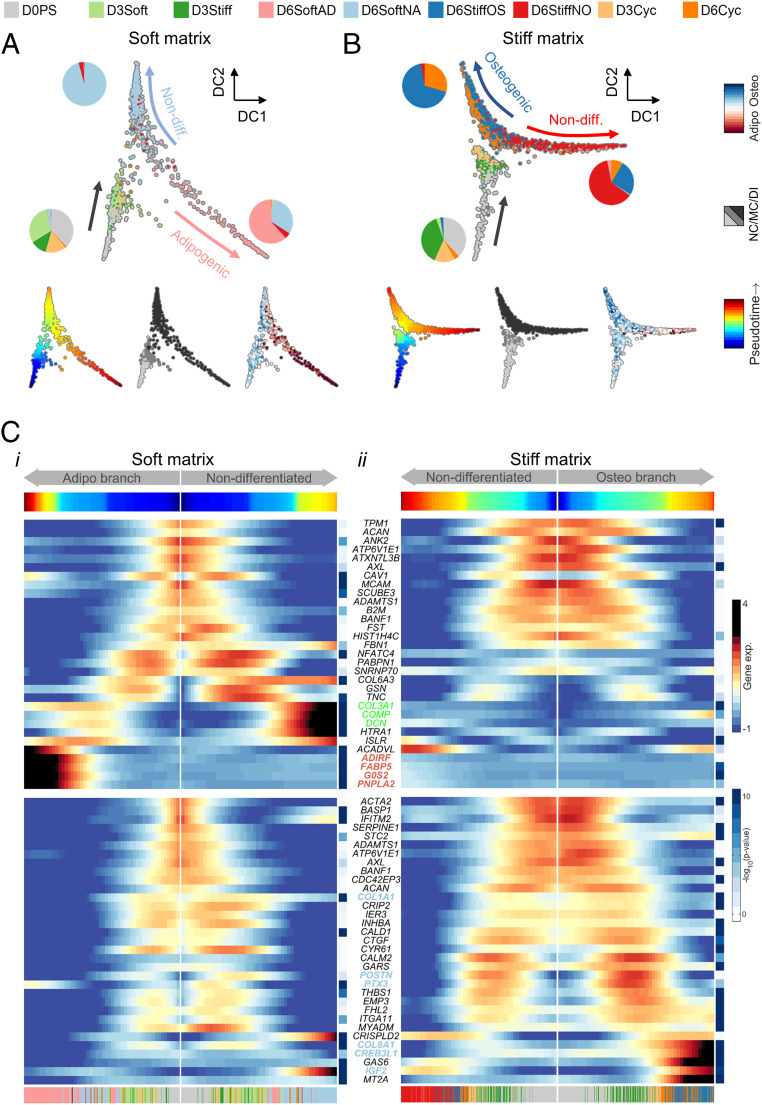
Diffusion pseudotime dynamics characterizes matrix-directed cell fate decision-making. (*A* and *B*) Diffusion mapping characterizes MSC propagation to matrix-conditioned states and bifurcation between (*A*) adipogenic and nonadipogenic fates on soft matrix and (*B*) osteogenic and nonosteogenic fates on stiff matrix. (*C*) Pseudotime projections of differentially expressed genes (adjusted *P* value < 10^−5^) that were upregulated in (*i*) cells cultured on soft matrix (*Top*) and (*ii*) in cells cultured on stiff matrix. Endochondral ossification (green) and adipogenic (red) markers are upregulated along soft matrix branches, and osteogenic markers (blue) are upregulated on a stiff matrix branch. NC: nonconditioned, MC: matrix conditioning, and DI: differentiation induction.

The single-cell transcriptomes provide multicellular snapshots of matrix-directed cell fate decision-making that highlight cell-to-cell variation. To reconstruct the effective propagation to matrix conditioning and early differentiation, we employed diffusion pseudotime analysis, which measures random-walk transcriptomic distances between cell states (Fig. 3 *A* and *B*) (49, 50). Cells propagated from D0PS to D3Soft or D3Stiff states and bifurcated between adipogenic and nonadipogenic fates on soft matrix. On stiff matrix, the bifurcation was between osteogenic and nonosteogenic fates. Upstream of bifurcation, the pseudotime propagation rate appears to be slower on stiff matrix than soft matrix. Differences in pseudotime rates suggest that during matrix conditioning, soft matrices support induction of expression of adipogenic genes concomitantly with the suppression of osteogenic genes that are continuously expressed in nonconditioned MSCs at low levels (41). On the soft matrix, cell cycling (D3Cyc) occurred concomitantly with matrix conditioning (Fig. 3*A*). Single-cell transcriptomes that expressed high levels of the cell cycling gene marker KI67 were temporally localized to early pseudotime stages that preceded bifurcation and CEBPA expression (*SI Appendix*, Fig. S4 *A*, *i*). However, on the stiff matrix, D6Cyc paralleled D6StiffOS, indicating that cell cycling propagated along matrix conditioning and early differentiation stages (Fig. 3*B*). Consistently, cells expressing high KI67 propagated toward the osteogenic branch and paralleled ATF4-expressing cells (*SI Appendix*, Fig. S4 *A*, *ii*). The association between cell cycling and preosteoblast differentiation was further supported by immunostaining of early differentiating MSCs on glass (Day 6) with antibodies targeting KI67 together with the osteogenic and adipogenic differentiation markers ATF4 and CEBPA (*SI Appendix*, Fig. S4 *B*, *i*). Cells that expressed high nuclear KI67 also expressed high nuclear ATF4 but not CEBPA. Cells in which KI67 was not localized to the nucleus expressed low ATF4 levels (*SI Appendix*, Fig. S4 *B*, *i*). Statistical analysis of a large number of cells revealed a linear correlation of nuclear ATF but not CEBPA with nuclear KI67 (*SI Appendix*, Fig. S4 *B*, *ii*). Hence, both single-cell transcript-level and protein-level analyses reveal an association between cell cycling and the differentiation of MSCs into preosteoblastic progenitors in line with previous reports (51, 52).

We next used the principle elastic tree algorithm to evaluate the effective transcriptome date. The Y-shaped scaffold trees are divided into a nonconditioned state and matrix-conditioned branch, adipogenic (soft matrix) and nonosteogenic (stiff matrix) branches, and nonadipogenic (soft matrix) and osteogenic (stiff matrix) branches with support nodes defined (*SI Appendix*, Fig. S5*A*). Pseudotime trajectories of ECM remodeling and cell adhesion genes that were highly expressed in expanded cells on polystyrene remained highly expressed during matrix conditioning on stiff matrices (Fig. 3 *C*, *i* and *ii*). Soft-matrix adipogenic and nonadipogenic branches were characterized by upregulation of adipogenic gene markers and endochondral ossification gene markers, respectively (Fig. 3 *C*, *i*, *Top*, red and green). The expression trajectory of the master regulator of adipogenesis *CEBPA* monotonically increased along the SoftAD branch and was retarded along the StiffNO branch (*SI Appendix*, Fig. S5 *B*, *i*). Suppression of *CEBPA* expression by stiffness cues further attenuated expression of downstream adipogenic markers including *G0S2*, *LPL*, and *ADIPOQ*. We detected upregulation of endochondral ossification gene markers during matrix conditioning of nonadipogenic cells on soft but not on stiff matrix (*SI Appendix*, Fig. S5 *B*, *ii*). The stiff-matrix osteogenic branch was characterized by upregulation of osteogenic gene markers (Fig. 3 *C*, *ii*, blue), which paralleled activation of SRF target genes that mediate mechanical cues and direct cell differentiation (*SI Appendix*, Fig. S5*C*) (53, 54).

### Identification of Matrix-Responsive Genes.

Bifurcation of the diffusion pseudotime maps highlights matrix-directed adipogenic and osteogenic differentiation by fat- and osteoid-like elasticities. Matrix-responsive genes would thus be characterized by the divergence of their pseudotime expression trajectories between SoftAD and StiffOS branches. To identify genes that were statistically significantly responsive to matrix, we first discarded genes with permutation test *P* values greater than 0.01. The remaining genes were scored for matrix-responsiveness (MR) by the area enclosed between SoftAD and StiffOS intensity profiles normalized by mean intensities:

$$\begin{array}{l} MR\left(g_{i}\right)=\underset{t}{\sum }\frac{\left|SA_{g_{i}}\left(t\right)-SO_{g_{i}}\left(t\right)\right|}{SA_{g_{i}}\left(t\right)+SO_{g_{i}}\left(t\right)}\Delta t. \end{array}$$ [1]

**Fig. 4.**
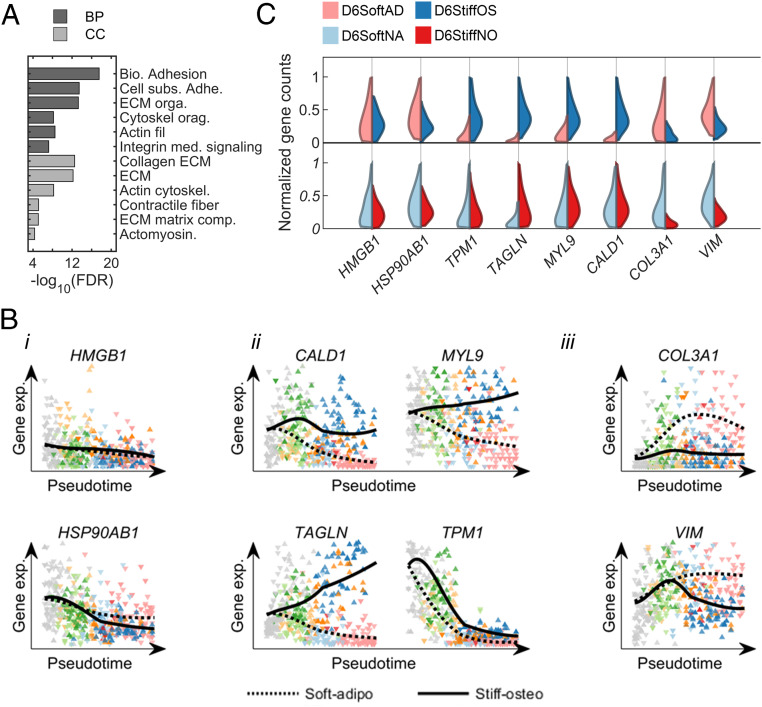
Matrix-responsive genes identified using pseudotime trajectories. (*A*) GO terms enriched in the top 100 matrix-responsive genes. (*B*) Pseudotime expression trajectories are shown for (*i*) representative housekeeping genes, (*ii*) highly ranked matrix-responsive genes that are upregulated by matrix stiffness, and (*iii*) highly ranked matrix-responsive genes that are upregulated by matrix softness. (*C*) Single-cell distributions of upregulation of cytoskeletal genes by matrix stiffness in matrix-directed differentiating subpopulations (*Top*) and nondifferentiating subpopulations (*Bottom*). BP: Biological process and CC: Cellular component.

Here, $SA_{g_{i}}\left(t\right)$ and $SO_{g_{i}}\left(t\right)$ are the intensities of gene $g_{i}$ along the SoftAD and StiffOS projections, respectively. The top-ranked genes were enriched for those involved in ECM remodeling, matrix adhesion, and actomyosin cytoskeletal organization (Fig. 4*A*). A list of the top 100 matrix-responsive genes is provided in Dataset S2.

Unlike housekeeping genes *HSP90AB1* and *HMGB1*, which showed no matrix-dependence (Fig. 4 *B*, *i*), genes that were upregulated on stiff matrices both during matrix conditioning and during early differentiation are enriched for actin-binding cytoskeletal components that belong to the so-called cellular contractome (55) and that are regulated by the SRF mechanotransduction signaling pathway (56). This included *CALD1* and *MYL9*, which encode proteins that regulate myosin head ATPase activity, *TPM1* that regulates actin–myosin interactions, and *TAGLN*, which encodes an actin cross-linker (Fig. 4 *B*, *ii*). *THY1* is also upregulated on stiff matrices; the protein it encodes directs osteogenesis (57, 58). *COL3A1*, which is a marker of endochondral ossification, and *VIM*, which encodes a type-III intermediate filament that is expressed in mesenchymal cells and contributes to adipogenesis (59), are both upregulated on soft matrices, (Fig. 4 *B*, *iii*).

### TPM1 Mediates Matrix-Directed Cell Fate Decisions.

**Fig. 5.**
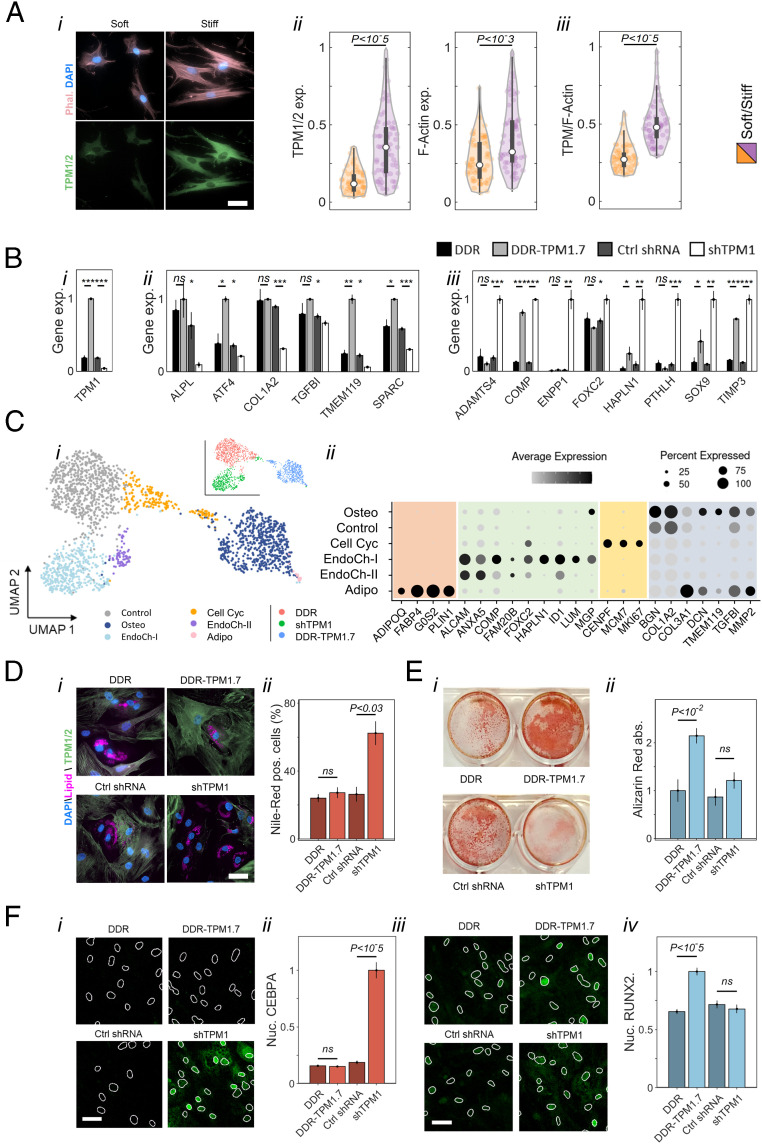
TPM1 mediates matrix-directed cell differentiation. (*A*, *i*) Immunofluorescence staining of matrix-conditioned MSCs (Day 3, male donor age 32) shows upregulation of TPM expression on stiff matrices concomitantly with F-actin polymerization. (Scale bar: 50 μm.) (*A*, *ii*) Quantitative analysis of immunofluorescence of tropomyosin (*Left*) and F-actin polymerization (*Right*) on stiff matrices. (*A*, *iii*) The distribution of single-cell ratios between tropomyosin expression and F-actin polymerization on soft and stiff matrix. (*B*) MSCs (male donor age 32 and female donor age 43) were transduced with constructs encoding Dendra2-conjugated TPM1.7 (DDR-TPM1.7), DDR control (DDR), short hairpin targeting TPM1 (shTPM1), or nonhairpin control sequence (Control shRNA), cultured for 3 d in basal medium, and profiled via population-level RNA sequencing. (*i*) Fourfold changes in TPM1 expression in transduced cells relative to controls altered the transcription of (*ii*) osteogenic and (*iii*) early endochondral ossification gene markers. Error bars represent standard deviation (STD) across three biological replicates. (*C*, *i*) Unsupervised clustering of single-cell transcriptomes of TPM1.7, shTPM1, and DDR Control MSCs (two female donors age 43 and 35) that were cultured for 3 d in basal medium and 3 d in bipotential medium is presented on a Uniform Manifold Approximation and Projection (UMAP) field. (*C*, *ii*) Fraction of positive cells and average expression of adipogenic, early endochondral ossification, cell cycling, and osteogenic gene markers are shown for the clustered subpopulations. (*D*, *i*) Nile-red staining of neutral lipids droplets formed in TPM1.7, shTPM1, and the corresponding controls after culture of 3 d in basal medium and 7 d in adipogenic medium. (Scale bar: 50 μm.) (*D*, *ii*) The percentage of cells that express lipid droplets stained with Nile-red and exceeding half nucleus projected area is highest in shTPM1 cells. (*E*, *i*) Alizarin-red staining of calcification by TPM1.7, shTPM1, and the corresponding controls after culture of 3 d in basal medium and 14 d in osteogenic medium. (*E*, *ii*) Alizarin-red absorbance demonstrates enhanced calcification by TPM1.7 cells. Error bars in *D* and *E* correspond to STD across three technical replicates. MSCs were derived from a male donor age 32. (*F*, *i*) CEBPA and (*F*, *iii*) RUNX2 immunostaining of TPM1.7, shTPM1, and the corresponding Control MSCs (female donor age 35) were performed after culture of 3 d in basal medium and 14 d in bipotential induction medium. (Scale bars: 20 μm.) Average nuclear intensities of (*F*, *ii*) CEBPA and (*F*, *iv*) RUNX2 are compared between tropomyosin-overexpressing, knocked-down, and Control MSCs. Error bars correspond to STD across nuclei.

The expression of the top-scored matrix-responsive genes that encode proteins involved in actin binding is more sensitive to matrix stiffness across progenitor cells that have the capacity to differentiate toward fat and bone (D6SoftAD and D6StiffOS, Fig. 4 *C*, *Top*) than in cells that exhibit impaired matrix-directed differentiation even in bipotential induction medium (D6SoftNA and D6StiffNO, Fig. 4 *C*, *Bottom*). *TPM1*, which was ranked 19, is of particular interest, as it encodes a protein that regulates myosin contractility on soft matrices (60). To study the regulation of *TPM1* by matrix elasticity, we performed quantitative immunofluorescence using an antibody that recognizes TPM1 and -2 isoforms. TPM1 was upregulated both at the RNA level (Fig. 4*C*) and at the protein level on stiff matrices concomitantly with increased filamentous actin (F-actin) polymerization (Fig. 5 *A*, *i* and *ii*). However, TPM1 expression was more sensitive to matrix elasticity than F-actin polymerization (Fig. 5 *A*, *iii*).

To study how TPM1 is involved in regulation of cell fate decisions, we designed lentiviral constructs encoding Dendra2 (DDR) conjugated to tropomyosin-1.7 isoform complementary DNA (cDNA) sequence (TPM1.7) and a short hairpin RNA (shRNA) complementary to *TPM1* (shTPM1) under control of a puromycin resistance selection sequence (*SI Appendix*, Fig. S6*A*). We also generated the respective DDR and nonhairpin shRNA control constructs. The extent of TPM1 overexpression and knockdown was fourfold at the RNA level (Fig. 5 *B*, *i*) and twofold at the protein level, as evaluated via quantitative immunofluorescence (*SI Appendix*, Fig. S6 *A*, *ii*) and Western blotting (*SI Appendix*, Fig. S6 *B* and *C*), relative to Control cells. To study the effects of tropomyosin knockdown and overexpression downstream of matrix elasticity, we cultured shTPM1, TPM1.7, and Control MSCs for 3 d in basal medium and performed population-level RNA sequencing. No adipogenic markers were activated at this stage under these conditions across three biological replicates. However, osteogenesis was distinctively activated in TPM1.7 cells (Fig. 5 *B*, *ii*), and early endochondral ossification genes were upregulated in shTPM1 cells (Fig. 5*B*, *iii*). Notably, the activation of early endochondral ossification genes in response to tropomyosin knockdown is reminiscent of the effects of matrix softness on the subpopulation of low–oxidative phosphorylation D3Soft cells (*SI Appendix*, Fig. S3 *A* and *B*).

To elucidate the transcriptional heterogeneity in response to tropomyosin knockdown and overexpression and the effects on cell fate decisions, we cultured TPM1.7, shTPM1, and Control MSCs for 3 d in basal medium and 3 d in bipotential medium and performed single-cell RNA profiling. Unbiased k-means clustering highlighted six subpopulations illustrated onto a dimensionally reduced Uniform Manifold Approximation and Projection graph (Fig. 5 *C*, *i*) (61). Most TPM1.7 cells cluster into one subpopulation (Osteo) that is characterized by activation of osteogenic gene markers (Fig. 5 *C*, *ii*). A subpopulation of cycling cells (Cell Cyc), which is likely associated with proliferating preosteoblastic progenitors similar to D6Cyc MSCs (51, 52), is shared between TPM1.7 and Control MSCs. Counter to TPM1.7 and Control MSCs, most shTPM1 cells are clustered into two subpopulations (EndoChon-I and -II). Both subpopulations are characterized by activation of early endochondral ossification genes (Fig. 5 *C*, *ii*). Importantly, no significant batch-driven contributions are observed as transcriptomes of the two donors overlapped within the specified clusters (*SI Appendix*, Fig. S2*D*). Despite the 3 d of culture in bipotential induction medium, no adipogenic gene markers were expressed, except for a minute contamination of preadipocytes (18 out of 2,024 cells). Consistent with the bifurcation of early differentiating MSCs on stiff matrix between osteogenic and nonosteogenic fates (Fig. 3*B*), RNA velocity maps of Control and TPM1.7 cells also exhibit a bifurcation between osteogenic and nonosteogenic fates (*SI Appendix*, Fig. S7 *A* and *B*) (62). Counter to Control and TPM1.7 MSCs, osteogenesis was suppressed in shTPM1 cells, thus eliminating a bifurcation dynamic (*SI Appendix*, Fig. S7*C*). Suppression of the osteogenic stem cell factors facilitated adipogenic lineage commitment on Day 10.

To assess tropomyosin effects on terminal differentiation of MSCs toward fat and toward bone, we cultured TPM1.7, shTPM1, and Control cells in basal medium for 3 d and in either adipogenic or osteogenic induction medium for 7 and 14 d, respectively. Adipogenesis and osteogenesis were assessed using Nile-red and Alizarin-red staining. Strikingly, we found that TPM1 knockdown increased adipogenesis 2.5-fold whereas TPM1.7 overexpression had no effect (Fig. 5 *D*, *i* and *ii*). Similarly, tropomyosin overexpression increased calcium deposition twofold whereas tropomyosin knockdown had no effect (Fig. 5 *E*, *i* and *ii*). The effects of tropomyosin on CEBPA and RUNX2 during MSC differentiation were further explored by culturing TPM1.7, shTPM1.7, and control MSCs in basal medium for 3 d and in bipotential induction medium for additional 2 wk. Adipogenic and osteogenic differentiation were assessed by quantifying the nuclear intensities of CEBPA (Fig. 5 *F*, *i*) and RUNX2 (Fig. 5 *F*, *iii*). Consistent with Nile-red and Alizarin-red signals, nuclear CEBPA increases in shTPM1 cells (Fig. 5 *F*, *ii*), and nuclear RUNX2 increases in TPM1.7 cells (Fig. 5 *F*, *iv*). Our measurements thus demonstrate the potent role of TPM1 in directing soft versus stiff tissue lineage commitment of MSCs in adipogenic, osteogenic, and bipotential media.

### Regulation of Tropomyosin-Directed MSC Differentiation.

**Fig. 6.**
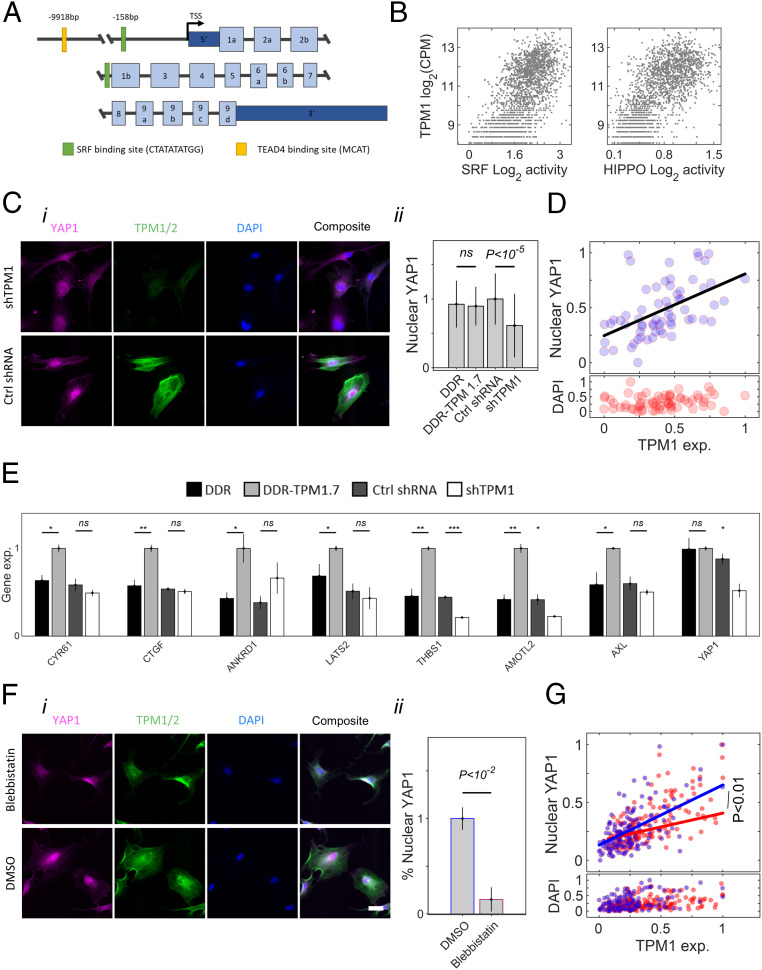
Tropomyosin-mediated regulation of mechanotransduced signaling pathways. (*A*) Schematic representation of *TPM1* genomic sequence. (*B*) Correlations of single-cell *TPM1* RNA levels with the average target genes of SRF (*Left*, Day 6 Spearman coefficient of correlation 0.62) and YAP1 (*Right*, Day 6 Spearman coefficient of correlation 0.31). (*C*, *i*) Immunofluorescence staining shows decreased nuclear localization of YAP1 in MSCs that express shTPM1 knockdown sequence compared with cells expressing a nonhairpin control sequence. (Scale bar: 50 μm.) (*C*, *ii*) Nuclear YAP1 levels are lowest in shTPM1 cells (*n* = 39 to 96 cells). MSCs derived from a male donor age 32. (*D*) Nuclear YAP1 increases linearly with tropomyosin levels (*n* = 70 wild-type cells). Error bars indicate STD. (*E*) Transcript levels of YAP1-target genes and YPA1 gene itself, as evaluated via total RNA sequencing, are upregulated in TPM1.7 cells and downregulated in shTPM1 cells compared with DDR and nonhairpin controls after culture of 3 d in basal medium. MSCs were derived from a male donor age 32 and a female donor age 43. (*F*, *i*) Nonconditioned primary MSCs (male donor age 21) were treated briefly (1 h) with blebbistatin or with DMSO and immunostained against tropomyosin-1,2 and YAP1. (*F*, *ii*) Percentage of cells expressing nuclear YAP1 decreases in response to blebbistatin-mediated cell relaxation. Error bars are STD across nuclei. (*G*) The increase in nuclear YAP1 in nonconditioned primary MSCs with tropomyosin is moderated in response to blebbistatin-mediated cell relaxation. Permutational multivariate analysis of variance (Permanova) statistics *P* values are evaluated.

The promoter-enhancer region of the *TPM1* gene contains an MCAT-binding element of TEAD 1 to 4 (transcription enhancer factors for YAP/TAZ) (63) and a CArG box that is recognized by SRF (Fig. 6*A*) (64, 65). Single-cell analysis revealed a significant positive correlation between *TPM1* expression and the expression of YAP1 and the SRF target genes (Fig. 6 *B*, *i* and *ii*) (26, 66). These data implicate TPM1 regulation by SRF and YAP1 downstream of matrix elasticity. F-actin stabilization was shown to activate noncanonical YAP signaling toward osteogenic differentiation (67). Hence, we tested whether TPM1 activates YAP1 signaling by comparing nuclear localization of YAP1 in shTPM1 with cells expressing a nonhairpin shRNA control (Fig. 6 *C*, *i*). Indeed, a twofold knockdown of TPM1 led to a 30% delocalization of YAP1 from the nucleus (Fig. 6 *C*, *ii*) (26). Consistently, nuclear YAP1 protein levels correlated with TPM1 protein levels in nonconditioned cells (Fig. 6*D*). To assess the effects of tropomyosin on the transcriptional activation of YAP1, we performed a population-level RNA sequencing of TPM1.7, shTPM1, and the corresponding control cells after 3 d in culture in basal medium (Fig. 6*E*). The expression of all YAP1 target genes was highest in TPM1.7 cells and lowest for most of the target genes in shTPM1 cells. Moreover, YAP1 itself was downregulated in shTPM1 cells. Our results are consistent with the effects of actin stress fiber assembly on nuclear localization of YAP1 and downstream gene regulation (26). To decouple the direct effects of tropomyosin and of cellular contractility, we performed a brief 1 h treatment of nonconditioned cells with blebbistatin, which is a pharmaceutical drug that blocks myosin contractility, thus eliminating significant changes in tropomyosin expression levels (Fig. 6 *F*, *i*). Cell relaxation lowered the fraction of cells with high nuclear YAP1 (Fig. 6 *F*, *ii*). In addition, blebbistatin-treated cells retained the increase in nuclear YAP1 with TPM1 levels similar to nontreated cells; however, the shallower slope suggests disruption of tropomyosin effects on YAP1 signaling due to cell relaxation (Fig. 6*G*).

## Discussion

The heterogeneity of primary MSCs has a profound impact on their clinical utility (7). It integrates multiscale contributions of the variation between donors, tissues of origin, clones, and single cells [as reviewed previously (4)]. Donor’s health condition and age are associated with a decline in MSC function, including self-renewal and differentiation capacities (68–71). However, variability is also observed between MSCs that were derived from the same tissue of origin of healthy donors of the same age (72). Hence, in addition to specifying tissue of origin, donor age, gender, and health condition, cellular indicators that characterize the functional heterogeneity of MSCs are necessary in order to compare between experiments and improve reproducibility. Here, we employed single-cell RNA sequencing for studying the heterogeneity of cell states within a population of bone marrow–derived MSCs during exposure to controlled mechanical signals and molecular factors that can induce soft versus stiff tissue lineage specification. Single-cell transcriptional profiling was broadly used for interrogating changes in cell states of multiple cell types during development (73, 74), tissue regeneration (75), tumorigenesis (76), immune response (77, 78), and cell differentiation (41). From single-cell analysis of MSCs cultured using the well-established matrix-directed conditioning and differentiation induction media, we identified nine cellular subpopulations using unsupervised clustering. MSC subpopulations are characterized by distinctive properties that are related to cell mechanosensitivity and differentiation capacity.

As expected, matrix softness supports adipocyte differentiation, and matrix stiffness promotes direct osteoblast differentiation. However, we also identified and characterized subpopulations of cells that failed to undergo osteogenesis or to upregulate adipogenic markers on stiff matrix and subpopulations of cells that failed to undergo adipogenesis or to upregulate osteogenic markers on soft matrix. This heterogeneity reflects a continuous phenotypic spectrum of bone marrow–derived stromal cells that lie between multipotent mechanosensitive progenitors that differentiate in tune with matrix cues, osteoblastic-committed cells that differentiate independent of matrix cues but no adipocytic-committed cells, and cells that lack the differentiation capacity to either lineage (*SI Appendix*, Fig. S1). In addition, we identified a subpopulation of MSCs that failed to undergo adipogenic differentiation in suitable induction media even when cultured on soft matrices but instead upregulated endochondral ossification genes (Figs. 2*C* and 5*C* and *SI Appendix*, Fig. S3).

Dynamic mapping of the cell fate decision processes provides means for screening matrix-responsive genes based on pseudotime trajectories. Of the average 3,800 expressed genes per cell, one of the most strongly matrix-dependent genes was *TPM1*, which encodes 12 alternatively spliced isoforms that form coiled-coil parallel dimers and copolymerize head-to-tail along actin filaments, thus regulating the interactions with myosin motors and actin-binding proteins (79), and permits high-frequency myosin power stroke cycles on stiff matrices (60). TPM1 antagonizes the inhibitory effects of the actin-severing proteins Cofilin and Gelsolin that negatively regulate YAP1 in mechanically relaxed cells (67) by inducing a nonfavorable conformational change to F-actin (80, 81). Via these interactions, tropomyosin decreases the mechanical flexibility of actin filaments (82, 83), protects stress fiber integrity, and stiffens the cell cortex (84), thus rendering mechanical strength under increasing load.

Mechanistically, *TPM1* can be transcriptionally regulated by the SRF and by other pathways that mediate mechanical cues via YAP1 as facilitated by the CArG box and MCAT-binding motifs, which is consistent with previous reports (54, 85–87). Our results indicate that upregulation of tropomyosin increases YAP1 nuclear localization and activation of TEAD-target genes including TPM1 (87), which directs osteogenic differentiation (26, 28). Downregulation of TPM1 expression or inhibition of actomyosin contractility similarly suppresses osteogenic differentiation. This further indicates that tropomyosin effects are mediated via stabilization of F-actin stress fibers (67). In this manner, the osteogenic stem cell factors are not upregulated in tropomyosin-regulated cells or in mechanically relaxed cells, thus alleviating the inhibition of adipogenic gene regulators (41). Expression of the adipogenic markers appears not to commence even in TPM1 knocked-down cells after 3 d of culture in basal medium nor after 3 additional d in bipotential medium. Instead, we detect the upregulation of early endochondral genes. Indeed, chondrogenic genes were upregulated in response to nuclear de-location of YAP1 after 3 d of culture on soft matrices (4 kPa) (88), and chondrogenesis was associated with YAP1 de-activation both in vitro and in vivo (89, 90). Adipogenic differentiation, which is interlinked with chondrogenic differentiation (91), is observed only after 1 wk of culture both in bipotential and in adipogenic induction media.

Collectively, we elucidate transcriptional variation in primary MSCs in which TPM-1 is identified as a matrix-responsive gene that is upregulated by matrix stiffness in early differentiating cells. However, cells that fail to differentiate in tune with matrix elasticity under the same culture conditions fail to regulate TPM1 in a matrix-dependent manner (Fig. 4*C*). In turn, TPM1 contributes to the stabilization of actomyosin stress fibers and a contractile cell state that direct cell differentiation via YAP1-mediated signaling downstream of matrix stiffness.

## Methods

### Cell Harvesting and Culture.

De-identified bone marrow aspirates were collected from the iliac crest of healthy human donors for allogeneic transplantation under written consent and the approval of the local institutional Helsinki Committee (0626-15-HMO). Aspirates were passed through a nylon cell strainer, separated by a Ficoll–Hypaque density gradient (LymphoprepTM, Alere Technologies), and resuspended in low-glucose Dulbecco's Modified Eagle Medium (DMEM; Biological Industries) supplemented with 1% L-glutamine (Biological Industries), 1% penicillin-streptomycin (Biological Industries), and 10% fetal bovine serum (Biological Industries). Cells were seeded into 75 cm^2^ culture flasks (30 × 10^6^ cells per flask) and cultured at 37 °C in a humidified atmosphere with 5% CO_2_. Cells were expanded and passaged once before seeded on the elastic matrices (P1). Medium was replaced twice weekly, and cell density was maintained <80 to 85% confluence. The positive (CD73 and CD90) and negative (HLA-DR, CD56, CD3, and CD45) surface marker repertoire was validated by fluorescence-activated cell sorting (FACS) using targeting antibodies (eBioscience).

### Adipogenic and Osteogenic Differentiation.

Adipogenic and osteogenic differentiation experiments and control conditions were performed as specified in the main text starting with 5,000 nonconditioned cells/cm^2^. Adipogenic induction was performed in low-glucose DMEM supplemented with 10 µg/mL^−1^ insulin, 500 mM 3-isobutyl-1-methylxanthine (Sigma-Aldrich), 1 µM dextran (Sigma-Aldrich), and 100 µM indomethacin (Sigma-Aldrich). Osteogenic induction was performed in low-glucose DMEM supplemented with 50 µg mL^−1^ L-ascorbic acid 2-phosphate (Sigma-Aldrich), 10 mM glycerol 2-phosphate (Sigma-Aldrich), and 10 nM dexamethasone (Sigma-Aldrich). Bipotential induction medium was prepared by mixing adipogenic and osteogenic induction media at equal volumes as reported (35, 36).

### Hydrogels with Controlled Elasticity.

Cells were cultured on thin polyacrylamide hydrogel films of 2 kPa (soft) and 25 kPa (stiff) elasticities covalently coated with rat tail type I collagen and attached to the bottom of 15 cm culture plates, 12-well plates, and 35 mm glass coverslips (Petrisoft, Matrigen).

### Western Blot.

Cells were harvested from 75 cm^2^ flasks using Trypsin, centrifuged at 300 G for 5 min to remove supernatant, washed in phosphate-buffered saline (PBS), and resuspended in ice-cold Nonidet P-40 lysis buffer: 150 mM NaCl, 1% Nonidet P-40, and 50 mM Tris HCl pH8.0 containing protease inhibitor mixture (APExBIO). Whole-cell lysates were then centrifuged for 20 min at 15,000 G, and the supernatant was transferred to a new tube. The total protein concentrations of the whole-cell lysates were quantified by Bradford Assay (Sigma-Aldrich) and combined with 2× loading buffer: 4% sodium dodecyl sulfate (SDS), 10% 2-mercaptoethanol, 20% glycerol, and 0.125 M Tris⋅HCl. To denature the samples, lysates were heated to 100 °C for 5 min. Protein separation was performed by sodium dodecyl sulfate-polyacrylamide gel (SDS-PAGE) electrophoresis. A total of 10 µg total protein of each sample was loaded onto a 4 to 20% Gebagel (Geba) and transferred onto a polyvinylidene fluoride (PVDF) membrane (Bio-Rad). After the transfer was complete, the membrane was washed in PBS and blocked at 4 °C overnight in a blocking buffer (Sigma-Aldrich). Immunofluorescent staining was performed by probing with primary antibodies anti-TPM1/2 antibody (TM311, Sigma-Aldrich) and anti-glyceraldehyde 3-phosphate dehydrogenase (GAPDH) antibody (Abcam) overnight at 4 °C. After rinsing the membrane twice in PBS for 15 min, the membrane was stained using secondary antibodies donkey anti-rabbit (Alexa Fluor 647; Abcam) and goat anti-mouse (Alexa Fluor 647; Abcam). Fluorescent images were visualized using a Typhoon phosphorimager (GE Healthcare). Protein-level quantification was done by ImageJ by calculating the average intensity of TPM1 bands after performing background subtraction. GAPDH intensity was used as used a loading control to normalize TPM1 levels.

### Immunofluorescence Sample Preparation and Imaging.

Cell samples were washed with PBS, fixed in 4% paraformaldehyde, blocked in 2% bovine serum albumin (BSA), permeabilized with 0.5% (vol/vol) Triton X-100 in PBS for 20 min, immersed in 2% BSA for 1 h, and rinsed in PBS. Staining using the primary antibodies targeting ATF4 and RUNX2 (Abcam), CEBPA (Santa Cruz), TPM1/2 (TM311, Sigma-Aldrich), and YAP1 (Proteintech), and the secondary antibodies donkey anti-mouse (Alexa Fluor 488; 1:100; Abcam), donkey anti-rabbit (Alexa Fluor 594), donkey anti-mouse (Alexa Fluor 647; 1:100; Abcam), and donkey anti-rabbit (Alexa Fluor 647; 1:100; Abcam) was performed according to manufacturer’s protocols. Samples were stained with 1 µg/mL^−1^ 4′,6-diamidino-2-phenylindole (DAPI, Sigma-Aldrich), dissolved in PBS, and immersed for 20 min and in 165 nM Phalloidin-iFluor 555 dissolved in PBS and immersed for 30 min. Lipid droplet staining was performed by immersing cells in 0.1 μg/mL^−1^ Nile-red (Sigma) dissolved in distilled water (DW) and immersed for 5 min. Immunofluorescence imaging was performed using a NIKON Ti-E inverted microscope equipped with an sCMOS iXon3 camera (Anodr) and a Spectra X light engine light source (Lumencor). A CFI Apo TIRF 60× Oil (Nikon) and a CFI Plan Apo VC 20× (Nikon) objective were used. Cell and nucleus projected areas were segmented and quantified using a custom-designed MATLAB code.

### Cell Culture Quantification of Adipogenesis and Osteogenesis.

Adipogenesis was quantified based on Nile-red and DAPI staining of fixed cells. The fraction of cells with Nile-red–stained neutral lipid area >50% of nucleus projected area (DAPI-stained) and with five distinct spherical shaped droplets or more was evaluated out of the total number of cells. Osteogenesis was quantified based on calcium deposition as evaluated via absorbance measurements (410 nm) of Alizarin-red–stained calcium deposits. Specifically, Alizarin-red was dissolved in double-distilled water (2% wt/vol) and HCl pH 4.2 adjusted. Fixed samples were rinsed in PBS, immersed in Alizarin-red solution for 15 min at room temperature, and rinsed twice with double-distilled water. Alizarin-red S-calcium complexes were extracted by immersion in 0.5 N HCl/5% SDS (wt/vol) extraction solution. The concentration of the extracted stain was quantified by measuring the absorbance at 410 nm (SmartSpec 3000 spectrophotometer, Bio-Rad) and normalized by the number of cells in each sample as measured via Hoechst 33342 (Thermo Fisher Scientific) staining.

### TPM1 Knockdown and Overexpression.

Knockdown of *TPM1* was performed using the pLKO.1 plasmid lentiviral backbone (a kind gift from Bob Weinberg, Addgene plasmid #8453) either encoding an shRNA with sequence complementary to *TPM1* (shTPM1: 5′-CGG​AGA​GGT​CAG​TAA​CTA​AAT-3′) or a control nonhairpin insert (5′-CCG​CAG​GTA​TGC​ACG​CGT-3′). Selection of expressing cells was performed in the presence of 1 mg/mL^−1^ puromycin (Sigma-Aldrich) for 2 wk following viral infection and confirmed using mCherry fluorescence signal. TPM1.7 overexpression was performed by generating a human MSC-derived cDNA library. The *TPM1.7* sequence was amplified using dedicated primers, a sequence encoding a C-terminal DDR sequence was conjugated, and the fragment was cloned into a lenti-EFIα pEIGW expression vector. Control overexpression vector did not contain the *TPM1.7* sequence.

Lentiviral particles were generated using human embryonic kidney (HEK) 293T cells. HEK cells were seeded in 55 cm^2^ plates at 50% confluence. Transfer (10 µg), packaging (10 µg psPAX2, Addgene #12260), and envelope (6 µg pMD2.G, Addgene #12259) viral plasmids were diluted in 500 µL serum-free DMEM. Next, plasmids were mixed with 500 µL polyethylenimine (PEI, Sigma-Aldrich) dissolved in DMEM for a final ratio of 1:2.5 DNA to PEI. HEK cells were cotransfected by incubating with DNA–PEI complexes for 18 h. Medium was exchanged, supernatant was collected after 24 and 48 h and filtered (0.45 µm PVDF, Millex), and MSCs (passage 1 and 2) were infected and transduced with lentiviral particles.

### Relaxation of Cell Contractility via Blebbistatin.

The myosin ATPase inhibitor blebbistatin (Sigma-Aldrich) was dissolved in dimethyl sulfoxide (DMSO, Sigma-Aldrich). Cells were treated with 50 µM blebbistatin or with an equal volume of DMSO. Cell cultures were not exposed to light. Cells were fixed and immunostained after 60 min of incubation.

### Single-Cell and Population-Level mRNA Sequencing.

Experimental procedures and computational analyses of single-cell and population-level messenger RNA (mRNA) sequencing are described in detail in *SI Appendix*.

## Supplementary Material

Supplementary File

Supplementary File

Supplementary File

Supplementary File

## Data Availability

All single-cell and population-level RNA sequencing data have been deposited in the publicaly accessible Gene Expression Omnibus (GEO) database (GSE166824).

## References

[1] C. Nombela-Arrieta, J. Ritz, L. E. Silberstein, The elusive nature and function of mesenchymal stem cells. Nat. Rev. Mol. Cell Biol. 12, 126–131 (2011). 21253000 10.1038/nrm3049 PMC3346289

[2] M. Crisan., A perivascular origin for mesenchymal stem cells in multiple human organs. Cell Stem Cell 3, 301–313 (2008). 18786417 10.1016/j.stem.2008.07.003

[3] B. Sacchetti., No identical “mesenchymal stem cells” at different times and sites: Human committed progenitors of distinct origin and differentiation potential are incorporated as adventitial cells in microvessels. Stem Cell Reports 6, 897–913 (2016). 27304917 10.1016/j.stemcr.2016.05.011 PMC4912436

[4] C. M. McLeod, R. L. Mauck, On the origin and impact of mesenchymal stem cell heterogeneity: New insights and emerging tools for single cell analysis. Eur. Cell. Mater. 34, 217–231 (2017). 29076514 10.22203/eCM.v034a14 PMC7735381

[5] M. J. Whitfield, W. C. Lee, K. J. Van Vliet, Onset of heterogeneity in culture-expanded bone marrow stromal cells. Stem Cell Res. 11, 1365–1377 (2013). 24103495 10.1016/j.scr.2013.09.004

[6] A. I. Caplan, Mesenchymal stem cells: Time to change the name! Stem Cells Transl. Med. 6, 1445–1451 (2017). 28452204 10.1002/sctm.17-0051 PMC5689741

[7] A. Wilson, M. Hodgson-Garms, J. E. Frith, P. Genever, Multiplicity of mesenchymal stromal cells: Finding the right route to therapy. Front. Immunol. 10, 1112 (2019). 31164890 10.3389/fimmu.2019.01112 PMC6535495

[8] I. Mastrolia., Challenges in clinical development of mesenchymal stromal/stem cells: Concise review. Stem Cells Transl. Med. 8, 1135–1148 (2019). 31313507 10.1002/sctm.19-0044 PMC6811694

[9] D. E. Discher, P. Janmey, Y. L. Wang, Tissue cells feel and respond to the stiffness of their substrate. Science 310, 1139–1143 (2005). 16293750 10.1126/science.1116995

[10] V. Vogel, M. Sheetz, Local force and geometry sensing regulate cell functions. Nat. Rev. Mol. Cell Biol. 7, 265–275 (2006). 16607289 10.1038/nrm1890

[11] A. Athirasala, N. Hirsch, A. Buxboim, Nuclear mechanotransduction: Sensing the force from within. Curr. Opin. Cell Biol. 46, 119–127 2017). 28641092 10.1016/j.ceb.2017.04.004

[12] N. Wang, J. P. Butler, D. E. Ingber, Mechanotransduction across the cell surface and through the cytoskeleton. Science 260, 1124–1127 (1993). 7684161 10.1126/science.7684161

[13] J. S. Park., Mechanical regulation of glycolysis via cytoskeleton architecture. Nature 578, 621–626 (2020). 32051585 10.1038/s41586-020-1998-1 PMC7210009

[14] A. Buxboim, K. Rajagopal, A. E. Brown, D. E. Discher, How deeply cells feel: Methods for thin gels. J. Phys. Condens. Matter 22, 194116 (2010). 20454525 10.1088/0953-8984/22/19/194116 PMC2864502

[15] A. del Rio., Stretching single talin rod molecules activates vinculin binding. Science 323, 638–641 (2009). 19179532 10.1126/science.1162912 PMC9339221

[16] Y. Sawada., Force sensing by mechanical extension of the Src family kinase substrate p130Cas. Cell 127, 1015–1026 (2006). 17129785 10.1016/j.cell.2006.09.044 PMC2746973

[17] B. Geiger, J. P. Spatz, A. D. Bershadsky, Environmental sensing through focal adhesions. Nat. Rev. Mol. Cell Biol. 10, 21–33 (2009). 19197329 10.1038/nrm2593

[18] F. Kong, A. J. García, A. P. Mould, M. J. Humphries, C. Zhu, Demonstration of catch bonds between an integrin and its ligand. J. Cell Biol. 185, 1275–1284 (2009). 19564406 10.1083/jcb.200810002 PMC2712956

[19] J. C. Friedland, M. H. Lee, D. Boettiger, Mechanically activated integrin switch controls alpha5beta1 function. Science 323, 642–644 (2009). 19179533 10.1126/science.1168441

[20] S. Rakshit, Y. Zhang, K. Manibog, O. Shafraz, S. Sivasankar, Ideal, catch, and slip bonds in cadherin adhesion. Proc. Natl. Acad. Sci. U.S.A. 109, 18815–18820 (2012). 23112161 10.1073/pnas.1208349109 PMC3503169

[21] B. W. Benham-Pyle, B. L. Pruitt, W. J. Nelson, Cell adhesion. Mechanical strain induces E-cadherin-dependent Yap1 and β-catenin activation to drive cell cycle entry. Science 348, 1024–1027 (2015). 26023140 10.1126/science.aaa4559 PMC4572847

[22] A. Buxboim., Matrix elasticity regulates lamin-A,C phosphorylation and turnover with feedback to actomyosin. Curr. Biol. 24, 1909–1917 (2014). 25127216 10.1016/j.cub.2014.07.001 PMC4373646

[23] C. Guilluy., Isolated nuclei adapt to force and reveal a mechanotransduction pathway in the nucleus. Nat. Cell Biol. 16, 376–381 (2014). 24609268 10.1038/ncb2927 PMC4085695

[24] A. Tajik., Transcription upregulation via force-induced direct stretching of chromatin. Nat. Mater. 15, 1287–1296 (2016). 27548707 10.1038/nmat4729 PMC5121013

[25] C. Y. Ho, D. E. Jaalouk, M. K. Vartiainen, J. Lammerding, Lamin A/C and emerin regulate MKL1-SRF activity by modulating actin dynamics. Nature 497, 507–511 (2013). 23644458 10.1038/nature12105 PMC3666313

[26] S. Dupont., Role of YAP/TAZ in mechanotransduction. Nature 474, 179–183 (2011). 21654799 10.1038/nature10137

[27] B. Geiger, A. Bershadsky, R. Pankov, K. M. Yamada, Transmembrane crosstalk between the extracellular matrix–Cytoskeleton crosstalk. Nat. Rev. Mol. Cell Biol. 2, 793–805 (2001). 11715046 10.1038/35099066

[28] A. Buxboim, I. L. Ivanovska, D. E. Discher, Matrix elasticity, cytoskeletal forces and physics of the nucleus: How deeply do cells ‘feel’ outside and in? J. Cell Sci. 123, 297–308 (2010). 20130138 10.1242/jcs.041186 PMC2816180

[29] A. J. Engler, S. Sen, H. L. Sweeney, D. E. Discher, Matrix elasticity directs stem cell lineage specification. Cell 126, 677–689 (2006). 16923388 10.1016/j.cell.2006.06.044

[30] A. Buxboim., Coordinated increase of nuclear tension and lamin-A with matrix stiffness outcompetes lamin-B receptor that favors soft tissue phenotypes. Mol. Biol. Cell 28, 3333–3348 (2017). 28931598 10.1091/mbc.E17-06-0393 PMC5687034

[31] J. Swift., Nuclear lamin-A scales with tissue stiffness and enhances matrix-directed differentiation. Science 341, 1240104 (2013). 23990565 10.1126/science.1240104 PMC3976548

[32] P. N. Patel, C. K. Smith, C. W. Patrick Jr, Rheological and recovery properties of poly(ethylene glycol) diacrylate hydrogels and human adipose tissue. J. Biomed. Mater. Res. A 73, 313–319 (2005). 15834933 10.1002/jbm.a.30291

[33] M. Stolz., Dynamic elastic modulus of porcine articular cartilage determined at two different levels of tissue organization by indentation-type atomic force microscopy. Biophys. J. 86, 3269–3283 (2004). 15111440 10.1016/S0006-3495(04)74375-1 PMC1304192

[34] F. Guilak, L. G. Alexopoulos, M. A. Haider, H. P. Ting-Beall, L. A. Setton, Zonal uniformity in mechanical properties of the chondrocyte pericellular matrix: Micropipette aspiration of canine chondrons isolated by cartilage homogenization. Ann. Biomed. Eng. 33, 1312–1318 (2005). 16240080 10.1007/s10439-005-4479-7

[35] J. Fu., Mechanical regulation of cell function with geometrically modulated elastomeric substrates. Nat. Methods 7, 733–736 (2010). 20676108 10.1038/nmeth.1487 PMC3069358

[36] M. Guvendiren, J. A. Burdick, Stiffening hydrogels to probe short- and long-term cellular responses to dynamic mechanics. Nat. Commun. 3, 792 (2012). 22531177 10.1038/ncomms1792

[37] B. M. Friedrich, A. Buxboim, D. E. Discher, S. A. Safran, Striated acto-myosin fibers can reorganize and register in response to elastic interactions with the matrix. Biophys. J. 100, 2706–2715 (2011). 21641316 10.1016/j.bpj.2011.04.050 PMC3117187

[38] R. D. González-Cruz, V. C. Fonseca, E. M. Darling, Cellular mechanical properties reflect the differentiation potential of adipose-derived mesenchymal stem cells. Proc. Natl. Acad. Sci. U.S.A. 109, E1523–E1529 (2012). 22615348 10.1073/pnas.1120349109 PMC3386052

[39] A. M. Klein., Droplet barcoding for single-cell transcriptomics applied to embryonic stem cells. Cell 161, 1187–1201 (2015). 26000487 10.1016/j.cell.2015.04.044 PMC4441768

[40] E. Z. Macosko., Highly parallel genome-wide expression profiling of individual cells using nanoliter droplets. Cell 161, 1202–1214 (2015). 26000488 10.1016/j.cell.2015.05.002 PMC4481139

[41] A. Rauch., Osteogenesis depends on commissioning of a network of stem cell transcription factors that act as repressors of adipogenesis. Nat. Genet. 51, 716–727 (2019). 30833796 10.1038/s41588-019-0359-1

[42] H. Choi., G0/G1 switch gene 2 has a critical role in adipocyte differentiation. Cell Death Differ. 21, 1071–1080 (2014). 24583640 10.1038/cdd.2014.26 PMC4207475

[43] I. Hisa., Parathyroid hormone-responsive Smad3-related factor, Tmem119, promotes osteoblast differentiation and interacts with the bone morphogenetic protein-Runx2 pathway. J. Biol. Chem. 286, 9787–9796 (2011). 21239498 10.1074/jbc.M110.179127 PMC3058974

[44] X. Yang, G. Karsenty, ATF4, the osteoblast accumulation of which is determined post-translationally, can induce osteoblast-specific gene expression in non-osteoblastic cells. J. Biol. Chem. 279, 47109–47114 (2004). 15377660 10.1074/jbc.M410010200

[45] T. Murakami., Signalling mediated by the endoplasmic reticulum stress transducer OASIS is involved in bone formation. Nat. Cell Biol. 11, 1205–1211 (2009). 19767743 10.1038/ncb1963

[46] Z. Hamidouche., Priming integrin alpha5 promotes human mesenchymal stromal cell osteoblast differentiation and osteogenesis. Proc. Natl. Acad. Sci. U.S.A. 106, 18587–18591 (2009). 19843692 10.1073/pnas.0812334106 PMC2773973

[47] Y. Zhang, G. Marsboom, P. T. Toth, J. Rehman, Mitochondrial respiration regulates adipogenic differentiation of human mesenchymal stem cells. PLoS One 8, e77077 (2013). 24204740 10.1371/journal.pone.0077077 PMC3800007

[48] F. Long, Building strong bones: Molecular regulation of the osteoblast lineage. Nat. Rev. Mol. Cell Biol. 13, 27–38 (2011). 22189423 10.1038/nrm3254

[49] L. Haghverdi, M. Büttner, F. A. Wolf, F. Buettner, F. J. Theis, Diffusion pseudotime robustly reconstructs lineage branching. Nat. Methods 13, 845–848 (2016). 27571553 10.1038/nmeth.3971

[50] L. Haghverdi, F. Buettner, F. J. Theis, Diffusion maps for high-dimensional single-cell analysis of differentiation data. Bioinformatics 31, 2989–2998 (2015). 26002886 10.1093/bioinformatics/btv325

[51] A. Rutkovskiy, K. O. Stensløkken, I. J. Vaage, Osteoblast differentiation at a glance. Med. Sci. Monit. Basic Res. 22, 95–106 (2016). 27667570 10.12659/MSMBR.901142 PMC5040224

[52] M. Galindo., The bone-specific expression of Runx2 oscillates during the cell cycle to support a G1-related antiproliferative function in osteoblasts. J. Biol. Chem. 280, 20274–20285 (2005). 15781466 10.1074/jbc.M413665200 PMC2895256

[53] E. N. Olson, A. Nordheim, Linking actin dynamics and gene transcription to drive cellular motile functions. Nat. Rev. Mol. Cell Biol. 11, 353–365 (2010). 20414257 10.1038/nrm2890 PMC3073350

[54] Q. Sun., Defining the mammalian CArGome. Genome Res. 16, 197–207 (2006). 16365378 10.1101/gr.4108706 PMC1361715

[55] R. Zaidel-Bar, G. Zhenhuan, C. Luxenburg, The contractome–A systems view of actomyosin contractility in non-muscle cells. J. Cell Sci. 128, 2209–2217 (2015). 26021351 10.1242/jcs.170068

[56] J. T. Connelly., Actin and serum response factor transduce physical cues from the microenvironment to regulate epidermal stem cell fate decisions. Nat. Cell Biol. 12, 711–718 (2010). 20581838 10.1038/ncb2074

[57] A. Paine., Thy1 is a positive regulator of osteoblast differentiation and modulates bone homeostasis in obese mice. FASEB J. 32, 3174–3183 (2018). 29401595 10.1096/fj.201701379R PMC5956243

[58] A. K. Picke., Thy-1 (CD90) promotes bone formation and protects against obesity. Sci. Transl. Med. 10, eaao6806 (2018). 30089635 10.1126/scitranslmed.aao6806

[59] K. Teichert-Kuliszewska., Increasing vimentin expression associated with differentiation of human and rat preadipocytes. Int. J. Obes. Relat. Metab. Disord. 20 (suppl. 3), S108–S113 (1996). 8680470

[60] H. Wolfenson., Tropomyosin controls sarcomere-like contractions for rigidity sensing and suppressing growth on soft matrices. Nat. Cell Biol. 18, 33–42 (2016). 26619148 10.1038/ncb3277 PMC5296190

[61] L. McInnes, J. Healy, N. Saul, L. Großberger, UMAP: Uniform manifold approximation and projection. J. Open Source Softw. 3, (2018).

[62] G. La Manno., RNA velocity of single cells. Nature 560, 494–498 (2018). 30089906 10.1038/s41586-018-0414-6 PMC6130801

[63] S. Pasquet., Transcription enhancer factor-1-dependent expression of the alpha-tropomyosin gene in the three muscle cell types. J. Biol. Chem. 281, 34406–34420 (2006). 16959782 10.1074/jbc.M602282200

[64] M. Y. Lee., Smooth muscle cell genome browser: Enabling the identification of novel serum response factor target genes. PLoS One 10, e0133751 (2015). 26241044 10.1371/journal.pone.0133751 PMC4524680

[65] I. Livyatan, Y. Aaronson, D. Gokhman, R. Ashkenazi, E. Meshorer, BindDB: An integrated database and webtool platform for “reverse-ChIP” epigenomic analysis. Cell Stem Cell 17, 647–648 (2015). 26637941 10.1016/j.stem.2015.11.015

[66] Z. Meng., RAP2 mediates mechanoresponses of the Hippo pathway. Nature 560, 655–660 (2018). 30135582 10.1038/s41586-018-0444-0 PMC6128698

[67] M. Aragona., A mechanical checkpoint controls multicellular growth through YAP/TAZ regulation by actin-processing factors. Cell 154, 1047–1059 (2013). 23954413 10.1016/j.cell.2013.07.042

[68] S. A. Kuznetsov, M. H. Mankani, P. Bianco, P. G. Robey, Enumeration of the colony-forming units-fibroblast from mouse and human bone marrow in normal and pathological conditions. Stem Cell Res. 2, 83–94 (2009). 19383412 10.1016/j.scr.2008.07.007 PMC2753860

[69] J. Wang, L. Liao, S. Wang, J. Tan, Cell therapy with autologous mesenchymal stem cells-how the disease process impacts clinical considerations. Cytotherapy 15, 893–904 (2013). 23751203 10.1016/j.jcyt.2013.01.218

[70] O. Katsara., Effects of donor age, gender, and in vitro cellular aging on the phenotypic, functional, and molecular characteristics of mouse bone marrow-derived mesenchymal stem cells. Stem Cells Dev. 20, 1549–1561 (2011). 21204633 10.1089/scd.2010.0280

[71] K. Stenderup, J. Justesen, C. Clausen, M. Kassem, Aging is associated with decreased maximal life span and accelerated senescence of bone marrow stromal cells. Bone 33, 919–926 (2003). 14678851 10.1016/j.bone.2003.07.005

[72] D. G. Phinney., Donor variation in the growth properties and osteogenic potential of human marrow stromal cells. J. Cell. Biochem. 75, 424–436 (1999). 10536366

[73] J. Cao., The single-cell transcriptional landscape of mammalian organogenesis. Nature 566, 496–502 (2019). 30787437 10.1038/s41586-019-0969-x PMC6434952

[74] S. Kanton., Organoid single-cell genomic atlas uncovers human-specific features of brain development. Nature 574, 418–422 (2019). 31619793 10.1038/s41586-019-1654-9

[75] T. Gerber., Single-cell analysis uncovers convergence of cell identities during axolotl limb regeneration. Science 362, eaaq0681 (2018). 30262634 10.1126/science.aaq0681 PMC6669047

[76] E. Azizi., Single-cell map of diverse immune phenotypes in the breast tumor microenvironment. Cell 174, 1293–1308.e36 (2018). 29961579 10.1016/j.cell.2018.05.060 PMC6348010

[77] Q. Zhang., Landscape and dynamics of single immune cells in hepatocellular carcinoma. Cell 179, 829–845.e20 (2019). 31675496 10.1016/j.cell.2019.10.003

[78] D. M. Fernandez., Single-cell immune landscape of human atherosclerotic plaques. Nat. Med. 25, 1576–1588 (2019). 31591603 10.1038/s41591-019-0590-4 PMC7318784

[79] P. W. Gunning, E. C. Hardeman, P. Lappalainen, D. P. Mulvihill, Tropomyosin–Master regulator of actin filament function in the cytoskeleton. J. Cell Sci. 128, 2965–2974 (2015). 26240174 10.1242/jcs.172502

[80] N. Kis-Bicskei., Tropomyosins regulate the severing activity of Gelsolin in isoform-dependent and independent manners. Biophys. J. 114, 777–787 (2018). 29490240 10.1016/j.bpj.2017.11.3812 PMC5984974

[81] Z. Ostrowska-Podhorodecka, M. Śliwinska, E. Reisler, J. Moraczewska, Tropomyosin isoforms regulate cofilin 1 activity by modulating actin filament conformation. Arch. Biochem. Biophys. 682, 108280 (2020). 31996302 10.1016/j.abb.2020.108280 PMC7291848

[82] H. Kojima, A. Ishijima, T. Yanagida, Direct measurement of stiffness of single actin filaments with and without tropomyosin by in vitro nanomanipulation. Proc. Natl. Acad. Sci. U.S.A. 91, 12962–12966 (1994). 7809155 10.1073/pnas.91.26.12962 PMC45560

[83] H. Isambert., Flexibility of actin filaments derived from thermal fluctuations. Effect of bound nucleotide, phalloidin, and muscle regulatory proteins. J. Biol. Chem. 270, 11437–11444 (1995). 7744781 10.1074/jbc.270.19.11437

[84] I. Jalilian., Cell elasticity is regulated by the tropomyosin isoform composition of the actin cytoskeleton. PLoS One 10, e0126214 (2015). 25978408 10.1371/journal.pone.0126214 PMC4433179

[85] Z. Sun., Tenascin-C promotes tumor cell migration and metastasis through integrin α9β1-mediated YAP inhibition. Cancer Res. 78, 950–961 (2018). 29259017 10.1158/0008-5472.CAN-17-1597 PMC5901716

[86] C. Ragu., The serum response factor (SRF)/megakaryocytic acute leukemia (MAL) network participates in megakaryocyte development. Leukemia 24, 1227–1230 (2010). 20428204 10.1038/leu.2010.80

[87] M. Kim, T. Kim, R. L. Johnson, D. S. Lim, Transcriptional co-repressor function of the hippo pathway transducers YAP and TAZ. Cell Rep. 11, 270–282 (2015). 25843714 10.1016/j.celrep.2015.03.015

[88] W. Zhong., YAP-mediated regulation of the chondrogenic phenotype in response to matrix elasticity. J. Mol. Histol. 44, 587–595 (2013). 23543231 10.1007/s10735-013-9502-y

[89] A. Karystinou., Yes-associated protein (YAP) is a negative regulator of chondrogenesis in mesenchymal stem cells. Arthritis Res. Ther. 17, 147 (2015). 26025096 10.1186/s13075-015-0639-9 PMC4449558

[90] H. Goto., Loss of *Mob1a/b* in mice results in chondrodysplasia due to YAP1/TAZ-TEAD-dependent repression of SOX9. Development 145, dev159244 (2018). 29511023 10.1242/dev.159244

[91] S. Zhou, S. Chen, Q. Jiang, M. Pei, Determinants of stem cell lineage differentiation toward chondrogenesis versus adipogenesis. Cell. Mol. Life Sci. 76, 1653–1680 (2019). 30689010 10.1007/s00018-019-03017-4 PMC6456412

