# Supplementary material for: Delineating the heterogeneity of matrix-directed differentiation toward soft and stiff tissue lineages via single-cell profiling
Source: Proc Natl Acad Sci U S A. 2021 May 3;118(19):e2016322118. doi: 10.1073/pnas.2016322118 (PMC8126831; doi:10.1073/pnas.2016322118)
Supplement: Supplementary File [file pnas.2016322118.sapp.pdf]

PNAS
www.pnas.org

Supplementary Information for
Delineating the heterogeneity of matrix-directed differentiation towards soft and stiff tissue lineages via single-cell profiling.

Shlomi Brielle, Danny Bavli, Alex Motzik, Yoav Kan-Tor, Xue Sun, Chen Kozulin, Batia Avni, Oren Ram,* and Amnon Buxboim,*

Correspondence:
Oren Ram. Email: oren.ram@mail.huji.ac.il;
Amnon Buxboim. Email: amnon.buxboim@mail.huji.ac.il;

This PDF file includes:

Supplementary Methods Appendix
Figures S1 to S7
SI References

Other supplementary materials for this manuscript include the following:

Datasets S1 to S3

Supplementary Methods Appendix

Droplet-based single-cell RNA barcoding and library preparation

inDrop high-throughput single-cell labeling was performed as reported with minor adaptations.[1,2] Briefly, cells were trypsin-detached, washed in phosphate-buffered saline (PBS, Sigma-Aldrich) and maintained dissociated in suspension supplemented with 15% OptiPrep (Sigma-Aldrich). Cells (~1000 cells/μl) were loaded into a chilled syringe pump and injected into a custom-designed microfluidics device. Flow settings were adjusted to allow individual cells to become encapsulated with single hydrogel beads presenting cell-specific barcoding oligonucleotide primers inside 3-5 nL encapsulation mixture droplets that contain 20 U μL^{-1} reverse transcriptase (SuperScript III, Thermo Fisher Scientific), First-Strand Buffer (Thermo Fisher Scientific), 0.6% (v/v) IGEPAL CA-630 (Sigma-Aldrich), 1 mM dNTPs (New England Biolabs), 6.67 mM DTT (Thermo Fisher Scientific), 2700 units mL^{-1} murine RNase inhibitor (New England Biolabs), and 0.1 M Tris-HCl (pH 8.0)).[2] Primers consisted of a poly-T sequence to trap mRNA molecules, two 7-to-10 base cell-specific barcodes, 5-base unique molecular identifier (UMI) sequence, and a T7 promoter sequence. Droplets were collected, the primers were photo-cleaved by exposing the emulsion to UV (365 nm 10 mW cm^{-2}, BlackRay Xenon lamp) for 10 minutes at room temperature, and reverse transcription of trapped mRNA was performed at 50 ºC for 2 hours. Droplets were then demulsified by adding 1H,1H,2H,2H-perfluoro-1-octanol (Sigma Aldrich) dissolved in HFE-7500 (Novec) at 20% (v/v). Single-stranded DNA primers and primer dimers were digested using 1 unit μL^{-1} ExoI (New England Biolabs) and 1 U μL^{-1} HinFI (Thermo Fisher Scientific). mRNA:cDNA hybrids were purified using Agencout AMPure XP beads (Beckman Coulter Genomics) according to the manufacturer's instructions. Second-strand cDNA synthesis was performed using NEBNext Ultra II Non-Directional RNA Second Strand Synthesis Module (New England Biolabs) and amplified by *in vitro* transcription (HiScribe, New England Biolabs). RNA products were fragmented using Ambion RNA Fragmentation Reagents (AM8740, Thermo Fischer Scientific), purified using Agencout AMPure XP beads, and reverse-transcribed using random hexamer primers (SuperScript III, Thermo Fisher Scientific). Single-stranded DNA fragments were linearly amplified via 14 PCR cycles (HiFi HotStart ReadyMix, Kapa Biosystems).

Sequencing and processing of single-cell RNA data: Non-conditioned, matrix-conditioned and early differentiating cells

scRNA profiling of non-conditioned, matrix-conditioned and early differentiating cells was performed using MSCs that were obtained from a male donor, age 40, weight 73 kg, height 173 cm. Libraries were paired-end sequenced (PE5: 45 bp and PE7: 35 bp) on a NextSeq 500 platform using a High Output kit (75 cycles, Illumina). Sequences were demultiplexed using indices and barcodes, permitting ≤ 1 nucleotide alignment substitution. After removing low-complexity poly-

adenylated sequences, transcripts were aligned against an indexed reference genome (GRCh38.p12) using Bowtie 0.12.8, and gene intensities were evaluated based on UMI frequency. Cells with high representation of mitochondrial RNA (>25%) or low gene number (≤1,900 per 15,000 UMIs) were discarded. Mitochondrial and ribosomal genes were discarded as well as low-frequency genes that were detected in ≤ 5 cells. Finally, UMI count was down-sampled to 15,000 per cell, thus normalizing gene expression and minimizing batch-specific confounders.

Clustering MSC subpopulations

Computational and statistical analyses were performed using established protocols in R (R Foundation for Statistical Computing) and Matlab (Matlab 2019 Mathworks Ltd). We distinguished highly variable genes from technical noise as described previously by Brennecke and colleagues.[3] Outlier expression values were truncated by winsorizing, the squared coefficient of variation (CV^2) versus the mean, μ, was fitted by $\alpha/\mu + \beta$,[3] and highly variable genes were identified using the Chi-squared test (FDR < 10^{-3}). UMI counts (CPM) of highly variable genes were $\log_2(x+1)$ transformed and single-cell transcriptomes were dimension-reduced via PCA. The number of significant PCs was determined based on a screen test. Unsupervised cell clustering was performed using *k*-means clustering in the PCA space. The number of clusters was determined based on within-cluster sum of squares (Scree plot). Genes that were differentially expressed across clusters were assigned based on the corresponding log-fold ratios of expression levels and the adjusted two-tailed Wilcoxon rank-sum test *p*-values (Benjamini–Hochberg method). Functional enrichment analysis was performed using Gene Set Enrichment Analysis database.[4]

Diffusion pseudotime analysis

Diffusion mapping was performed using the Destiny package in R.[5] Only genes that varied significantly across soft and stiff matrix-conditioned cells, which we identified using the CV^2 plot as described above (FDR < 10^{-3}), were retained. Diffusion mapping was calculated separately for cells that were cultured on soft matrices (NV, D3Soft and D6Soft; Fig. 3a) and cells that were cultured on stiff matrices (NV, D3Stiff and D6Stiff; Fig. 3b). For each matrix, we performed PCA dimensionality reduction, and the diffusion components were calculated based on the top twenty principle components that maximized variance between transcriptomes with the local sigma calculated by nearest-neighbour approximation (k = 50).

Matrix-directed cell differentiation dynamics was analysed using the MeRLOT package in R.[6] Soft and stiff matrix scaffold trees were reconstructed using the first two diffusion components (CalculateScaffoldTree function in R). Principle elastic trees were calculated, and cells were assigned to 50 discrete support nodes that were aligned along the bifurcating branches

(CalculateElasticTree function in R). Setting the first support nodes that are associated with non-conditioned MSC state as the initial pseudotime, pseudotime values were further propagated along the bifurcating branches of the support trees in proportion with the distance from t_0 and cells were assigned accordingly (CalculatePseudotimes function in R). Next, cells were binned into pseudotime sets (25 bins for the trunk and for each of the branches), and dynamic gene expression profiles were averaged within each bin along the bifurcating trajectories of the soft and stiff matrix trees.[7] Gene trajectories were further smoothed by fitting a natural cubic spline with three degrees of freedom (Figs. 4a and Fig. S5b).

Gene trajectories along the Soft-Adipo and Stiff-Osteo were clustered using *k*-means, and the clusters were chronologically ordered according to the average pseudotime point of maximal gene expression. Genes that were differentially expressed between cell subpopulations (lfc > 1, adjusted FDR < 10^{-5}) were ranked by similarity using a cosine-distance metric within each cluster, and trajectories of most similar genes within each cluster were plotted with respect to SoftAD (Fig. 3b-i) and StiffOS (Fig. 3b-ii) branches.

Permutation test of mechanically diverging genes

The statistical significance of gene divergence along the SoftAD versus StiffOS trajectories was evaluated using a permutation test. Specifically, cells were randomly reassigned a branch label while maintaining the correct overall distribution of cells between branches. New branch curves were then calculated, and the normalized divergence area between the curves was calculated as described above. A distribution of the divergence area was obtained based on 1,000 random permutations, and *p*-value of the non-permutated divergence was calculated.

Pathway analysis

The activation of SRF and YAP1 signaling was evaluated by measuring the expression levels of respective target genes.[8,9] Single-cell expression levels of target genes showing a minimum average expression (UMI > 0.5) were linearly rescaled to [0,1], and signaling pathway activation was scored in each cell by the average normalized intensity.

Transcriptome-based scoring of adipogenic-osteogenic differentiation

Adipogenic and osteogenic differentiation was scored based on a subset of established markers (adipocytes: *PPARG*, *CEBPA*, *PLIN1*, *G0S2*; osteocytes: *COMP*, *TMEM119*, *DCN*, *BCAP31*, *ITGA5*, *COL1A1*, *ATF4*, *CREB3L1*, *FSTL3*) using:

$$r_c = \frac{1}{N_{osteo}} \sum_i^{osteo} \frac{g_{c,i}}{\max g_{c,i}} - \frac{1}{N_{adipo}} \sum_i^{adipo} \frac{g_{c,i}}{\max g_{c,i}} ,$$

where $g_{c,i}$ is the transcript level of gene *i* in cell *c*.

Transcriptome-based scoring of cell cycling

Cell cycling scoring of single-cell transcriptomes based on a combined group of gene markers of S phase (43 genes) and G2M phase (54 genes) as listed in Supplementary Dataset S3.[10] The single-cell expression intensities of these gene markers were z-scored normalized across cells and cell-cycling scores were obtained by averaging all gene markers per transcriptome. Cells in G1-phase will not express S phase nor G2M phase markers.

Transcriptome-based scoring of oxidative phosphorylation state

Oxidative phosphorylation was scoredusing a set of 23 genes that encode proteins of the electron transport chain (complexes I-V) and the ATP synapse complex. The single-cell expression intensities of these genes were z-score normalized and averaged. D3soft subpopulation was divided into low oxidative (< -0.23), mid (> -0.23 and > 0.13) and high (> 0.13) oxidative phosphorylation cells.

Sequencing and processing of single-cell RNA data: TPM1.7, shTPM1, and Control cells

scRNA profiling of TPM1.7, shTPM1 and control conditions was performed using MSCs that were isolated from two donors (Donor-1: Female age 43, weight 88 kg, height 179 cm. Donor-2: Female age 35). Cells were seeded at 5,000 cells cm^{-2} in polystyrene 75 cm^2 flasks and cultured in basal medium for three days and three days in bi-potential induction medium. Droplet-based scRNA seq was performed as described above. Low-complexity poly-adenylated sequences were filtered out, the remaining sequences were de-multiplexed using the dropEst pipeline,[11] and aligned to a human indexed reference genome (GRCh38.p12) using TopHat2 2.0.08.[12] Gene expression loom file was generated based on the aligned transcript UMIs that included both spliced and unspliced sequence counts. Cell quality control was performed using the R software package Seurat3 to remove transcriptomes with < 500 genes or < 1,000 unique UMIs or cells with >25% mitochondrial RNA.[13] Log-scaled gene expression intensities were normalized according to transcript number. PCA dimension reduction of the single-cell transcriptomes was performed with respect to the highly variable genes and the top twenty principal components were UMAP-visualized. Graph-based clustering was performed in the PCA space according to the Seruat3 pipeline.

RNA velocity analysis

RNA velocity was performed using the show.velocity.on.embedding.cor function in the velocyto package in R (La Manno et al., 2018). 35x35 grid RNA velocity fields were visualized based on the top two principal components of the single cell transcriptomes. Porjected arrows were square-root scaled.

Population-level RNA sequencing

Population-level RNA profiling was performed using MSCs that were isolated from two donors (Donor-1: Male age 32, 64 kg. Donor-2: Female age 43, weight 88 kg, height 179 cm). DDR-TPM1.7 (overexpressed), shTPM1 (knockdown), and DDR Control MSCs were expanded on polystyrene plates in basal medium. Cell extracts were extracted by treating cells with Trizol reagent (Bio-Labs) and homogenizing with a 25-gauge needle. Cell lysates were mixed with Chloroform (Sigma-Aldrich) in a 5:1 ratio. The aqueous phase was separated via centrifugation and purified using RNeasy Mini Kit (Qiagen) according to the manufacturer's protocol. Biological triplicates of poly(A) RNA were extracted and genomic libraries were generated using the KAPA Stranded mRNA-Seq Kit (KAPA Biosystems). Single-end sequencing with a 30 million reads depth per sample were performed using NextSeq500 (Illumina) with the High Output Kit v2 (Illumina). Human genome alignment (hg19/GRCh38) was performed using Bowtie2,[14] and unique reads were counted using the HTSeq package.[15] Differential gene expression analysis was performed with DESeq2.[16]

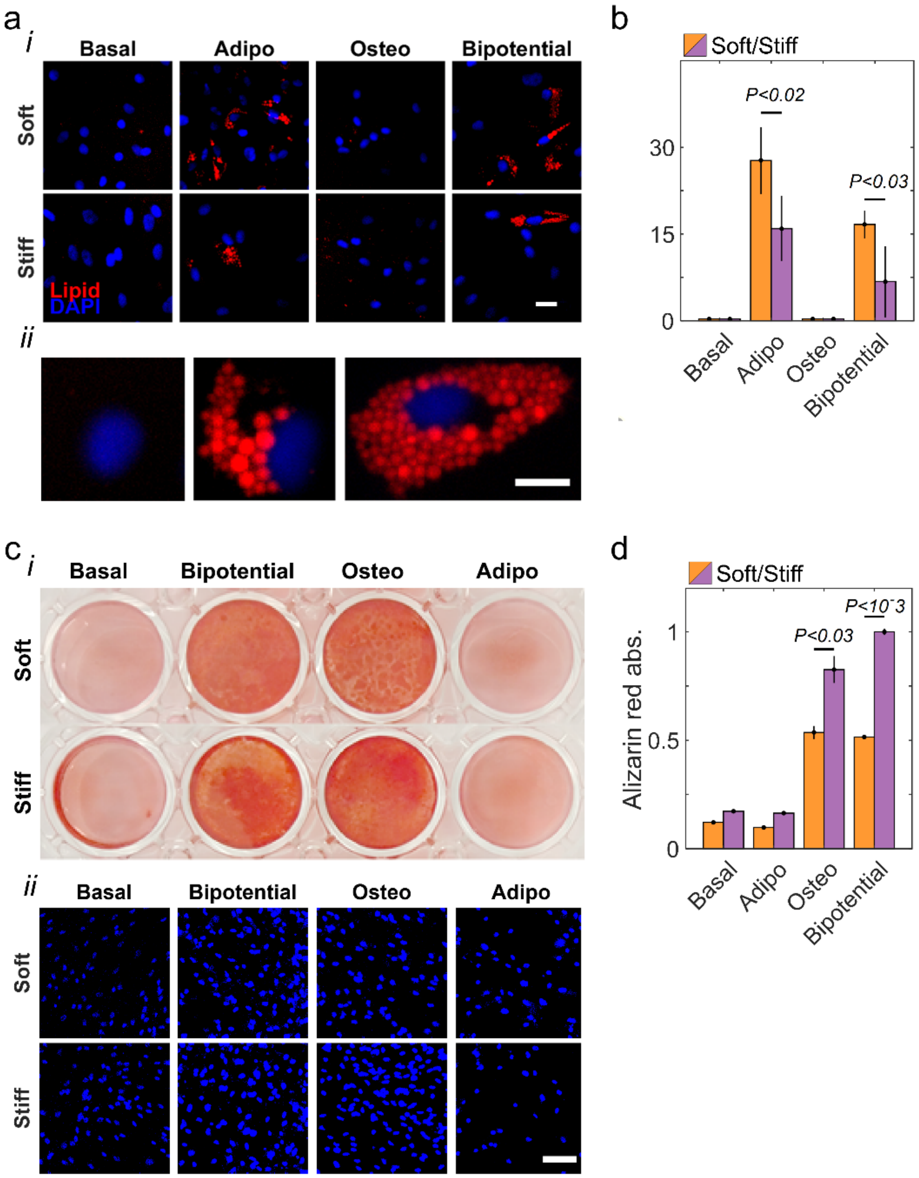

Figure S1 | Matrix elasticity directs MSC differentiation toward soft and stiff tissue lineages. **a,** (i) Adipogenic differentiation was evaluated via Nile red neutral lipid staining of cells that were cultured for 3 days in basal medium and 7 days in basal, adipogenic, osteogenic, or bi-potential induction media on soft and stiff matrices. (ii) In some cells no lipids formed where in other cells the area captured by the lipid droplets was comparable with nucleus projected area or above. Scale bar: 10 μm. (iii) The fraction of positively stained cells (> half nucleus area) was high on soft matrices only under medium conditions that support adipogenesis. **c,** (i) Osteogenic differentiation was evaluated via Alizarin red staining of calcification deposition by cells that were cultured for 3 days in basal medium and subsequent 14 days in basal, adipogenic, osteogenic, or bi-potential induction mediu on soft and stiff matrices. (ii) Cells maintained a homogenous coverage along two weeks of culture under all specified conditions as demonstrated via Hoechst staining. Scale bar: 50 μm. **d,** Osteogenic differentiation was evaluated by average Alizarin red intensities across three replica showing baseline level osteogenesis in basal and adipogenic media and upregulation on stiff matrices in osteogenic and bi-potential medium conditions. Error bars indicate STD. MSCs were derived from a male donor age 32.

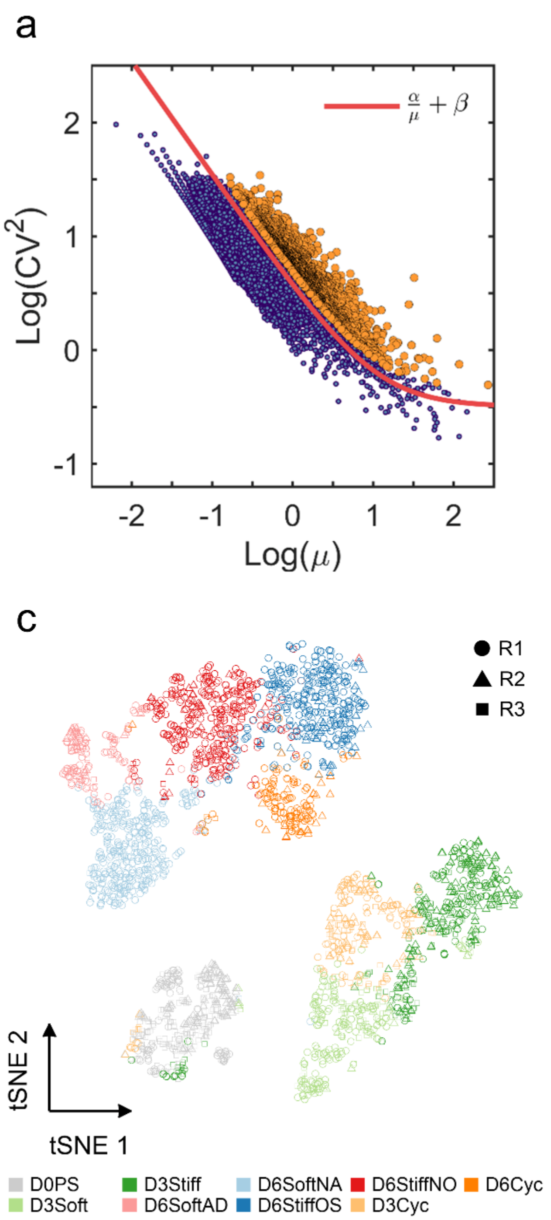

Figure S2 | Single-cell transcriptomes are clustered based on culture settings and matrix elasticities. **a,** Highly variable genes across non-conditioned, matrix-conditioned, and early-differentiation induction single-cell transcriptomes are detected using a CV^2-mean plot (orange). **b,** Single-cell transcriptomes cluster according to cell state (top left). MC (top right) and DI (bottom-left) cells cluster according to matrix elasticity. **c,** The technical replicates of NC, MC and DI cells (male donor age 32) are superimposed on the t-SNE map showing overlapping agreement across all conditions. **d,** The single-cell transcriptomes of donors D1 (female donor age 43) and D2 (female donor age 35) overlap on the UMAP field of TPM1.7, shTPM1 and Control conditions. NC: non-conditioned. MC: matrix conditioned. DI: differentiation induction.

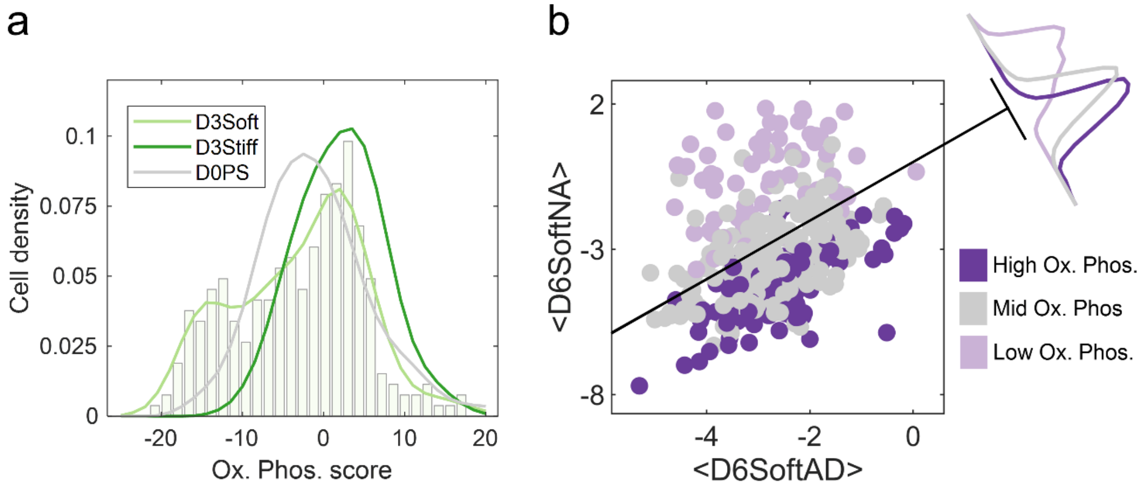

Figure S3 | Soft matrix supports low-oxidative phosphorylation cells directed towards endochondral ossification. **a,** Comparison between single cell distributions of D0PS cells and matrix-conditioned D3Soft and D3Stiff subpopulation reveal the emergence of low oxidative phosphorylation (OxPhos) cells in the D3Soft subpopulation. Bars correspond to binned values of D3Soft cells. **b,** Projection analysis of D3Soft cells onto the transcriptome averages of early differentiating soft-matrix subpopulations D6SoftAD and D6SiftND cells was calculated with respect to differentially expressed genes (flc>1; FDR<10^-5). Low oxidative-phosphorylation and high oxidative phosphorylation D3Soft cells appear to sub-cluster in tune with transcriptome projections onto D6SoftNA and D6SoftAD cells, respectively.

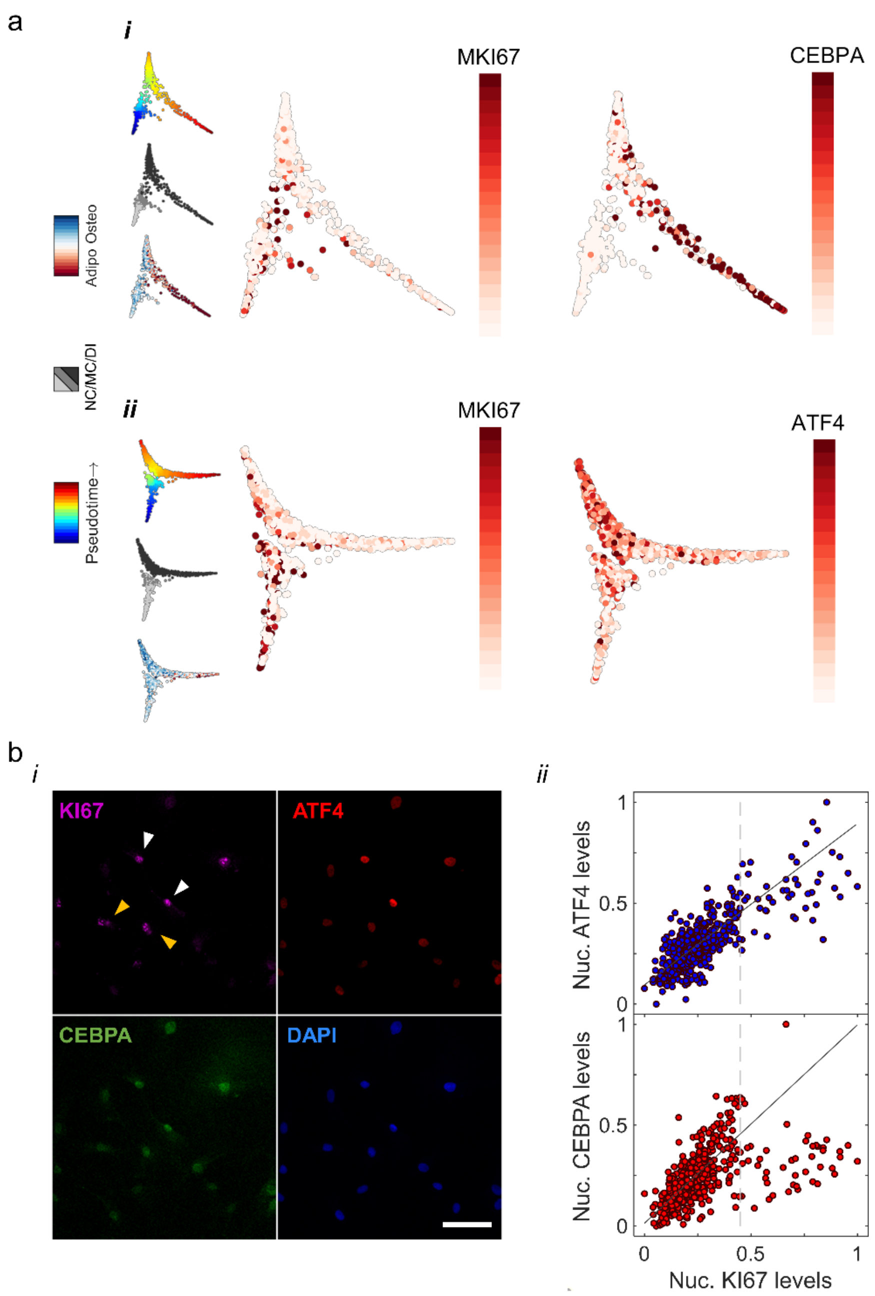

Figure-S4 | Cell cycling is linked with osteogenic linage commitment of pre-osteoblastic progenitors. a. (i) Early-differentiating MSCs (Day-6) were cultured on glass and immunostained against the gene markers of cycling marker (*KI67*), osteogenesis (ATF4), adipogenesis (*CEBPA*), and DAPI. High nuclear *KI67* cells (white arrowheads) express high nuclear *ATF4* but not nuclear *CEBPA* counter to low nuclear KI67 cells (yellow arrowheads). Scale-bar: 50 μm. **b.** Single-cell transcript levels of *MKI67* are correlated with (i) *CEBPA* and with (ii) *ATF4* on soft and stiff matrix pseudotime diffusion maps. Cell cycling is restricted to matrix-conditioned MSCs on soft matrix but extends towards early-differentiation osteogenic cells on stiff matrix. MSCs were derived from a male donor, age 40.

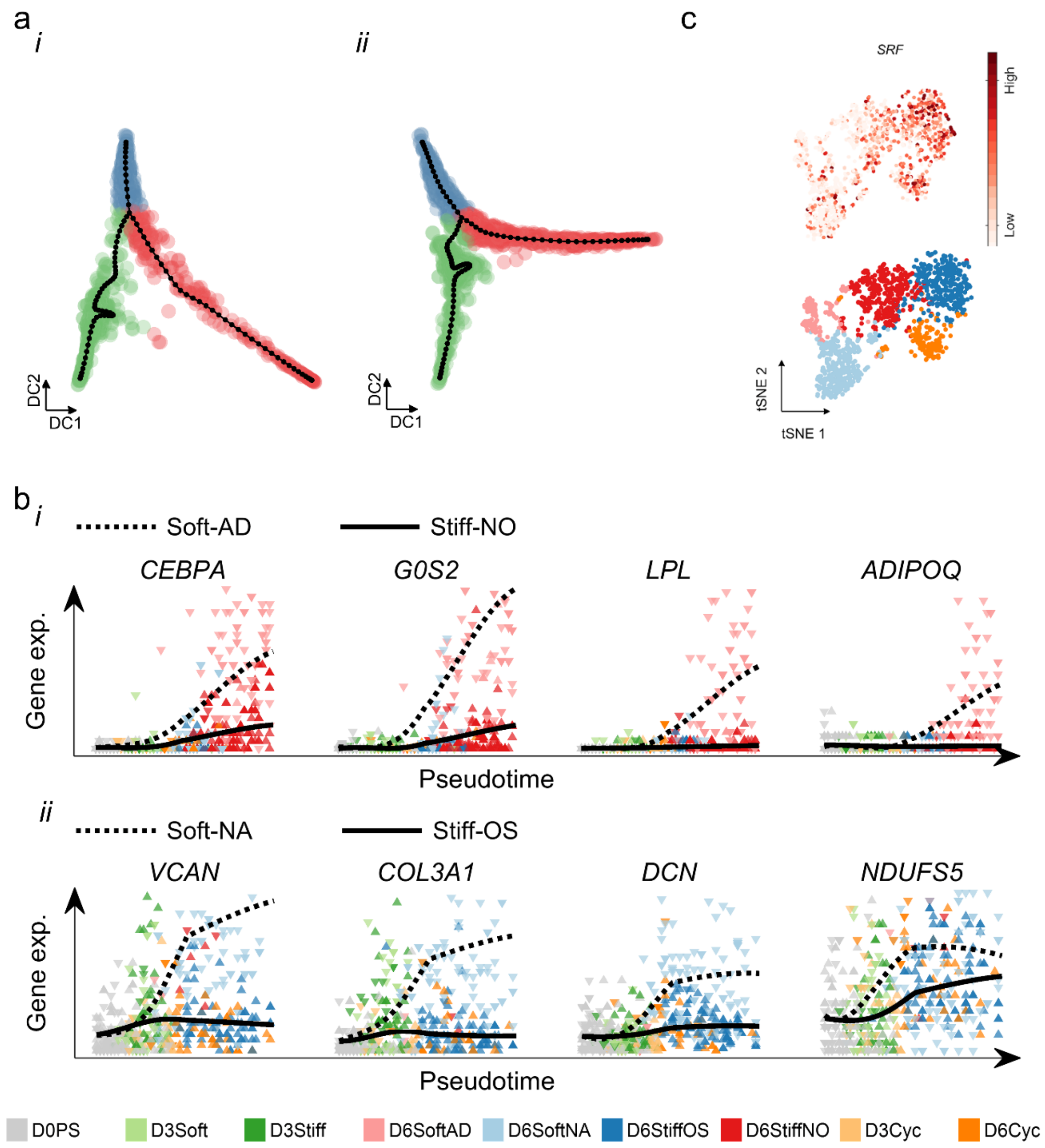

Figure S5 | Soft matrices direct adipogenesis of multipotent cells and support endochondral ossification of non-adipogenic cells. a, Scaffold tree branch assignments of matrix conditioning (green), adipogenic/non-osteogenic (red), and osteogenic/non-adipogenic (blue) fates on (i) soft and (ii) stiff matrices serve as templates for principle elastic tree support nodes (black). **b,** (i) Comparison between the pseudotime gene trajectories of non-osteogenic Day-6 subpopulations show upregulation of *CEBPA* and downstream adipogenic markers on soft matrices. (ii) Comparison between the pseudotime gene trajectories of non-adipogenic Day-6 subpopulations show upregulation of endochondral ossification and electron transport chain genes on soft matrix. **c,** Single-cell SRF activation map of early-differentiating subpopulations. tSNE map is reproduced from Fig. 1c.

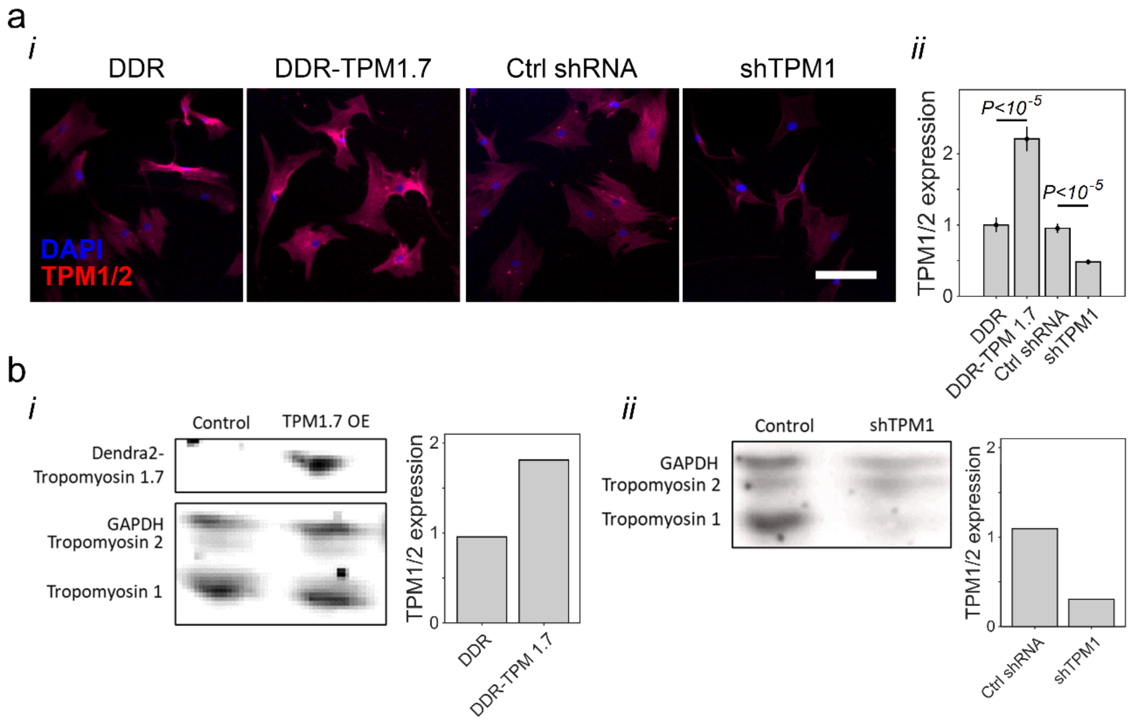

Figure S6 | Perturbing tropomyosin-1 expression in MSCs. a, (i) Representative images of cells transduced with vectors for overexpression of tropomyosin-1.7 isoform conjugated to Dednra2 fluorescent protein (DDR-TPM1.7), short hairpin silencing RNA targeting TPM1 gene (shTPM1), and the corresponding controls expressing Dendra2 (DDR) and non-hairpin control sequence (Ctrl shRNA). Cells were immunostained against tropomyosin isoforms. Scale bar: 100 μm. (ii) Quantitative immunofluorescence analysis of tropomyosin levels shows approximately twofold upregulation in TPM1.7 and twofold downregulation in shTPM1 cells compared with controls. **b,** Tropomyosin immunoblotting shows (i) ectopic overexpression above endogenous levels that increases protein levels twofold in TPM1.7 relative to DDR control, and (ii) twofold knockdown relative to Ctrl shRNA cells. Total protein levels were adjusted and normalized to GAPDH loading control. DDR: Dendra2 control. DDR-TPM1.7: Dendra2-conjugated tropomyosin 1.7. Ctrl shRNA: Non-hairpin insert sequence. shTPM1: shRNA targeting TPM1. MSCs were derived from a male donor, age 40.

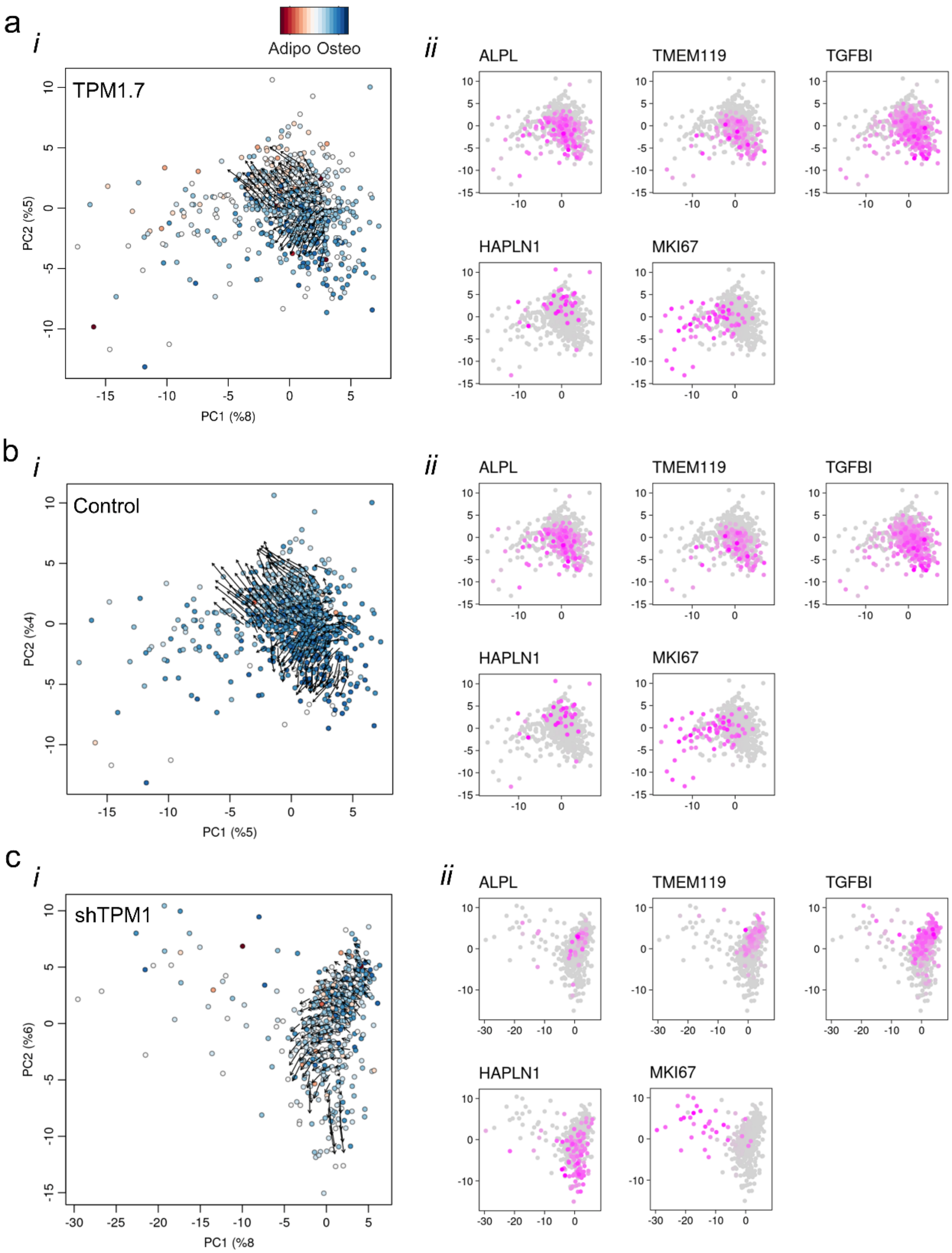

Figure-S7 | Bifurcation in RNA velocity fields is determined by tropomyosin-1 perturbations: RNA velocity fields of (a) Control and (b) TPM1.7 overexpressing MSCs show effective bifurcating transcriptional flow towards osteogenic and non-osteogenic fates, counter to (c) shTPM1 MSCs. Intensities of gene markers of osteogenesis (*ALPL*, *TMEM119*, and *TGFBI*), early endochondral ossification (*HPALN1*), and cell cycling (*MKI67*) are independently normalized within each condition. MSCs were derived from two female donors age 43 and 35.

SI References

1 Klein, A. M. *et al.* Droplet barcoding for single-cell transcriptomics applied to embryonic stem cells. *Cell* **161**, 1187-1201, doi:10.1016/j.cell.2015.04.044 (2015).

2 Zilionis, R. *et al.* Single-cell barcoding and sequencing using droplet microfluidics. *Nat Protoc* **12**, 44-73, doi:10.1038/nprot.2016.154 (2017).

3 Brennecke, P. *et al.* Accounting for technical noise in single-cell RNA-seq experiments. *Nat Methods* **10**, 1093-1095, doi:10.1038/nmeth.2645 (2013).

4 Subramanian, A. *et al.* Gene set enrichment analysis: a knowledge-based approach for interpreting genome-wide expression profiles. *Proc Natl Acad Sci U S A* **102**, 15545-15550, doi:10.1073/pnas.0506580102 (2005).

5 Angerer, P. *et al.* destiny: diffusion maps for large-scale single-cell data in R. *Bioinformatics* **32**, 1241-1243, doi:10.1093/bioinformatics/btv715 (2016).

6 Parra, R. G. *et al.* Reconstructing complex lineage trees from scRNA-seq data using MERLoT. *Nucleic Acids Res* **47**, 8961-8974, doi:10.1093/nar/gkz706 (2019).

7 Qiu, X. *et al.* Single-cell mRNA quantification and differential analysis with Census. *Nat Methods* **14**, 309-315, doi:10.1038/nmeth.4150 (2017).

8 Wang, Y. *et al.* Comprehensive Molecular Characterization of the Hippo Signaling Pathway in Cancer. *Cell Rep* **25**, 1304-1317 e1305, doi:10.1016/j.celrep.2018.10.001 (2018).

9 Rouillard, A. D. *et al.* The harmonizome: a collection of processed datasets gathered to serve and mine knowledge about genes and proteins. *Database (Oxford)* **2016**, doi:10.1093/database/baw100 (2016).

10 Macosko, E. Z. *et al.* Highly Parallel Genome-wide Expression Profiling of Individual Cells Using Nanoliter Droplets. *Cell* **161**, 1202-1214, doi:10.1016/j.cell.2015.05.002 (2015).

11 Petukhov, V. *et al.* dropEst: pipeline for accurate estimation of molecular counts in droplet-based single-cell RNA-seq experiments. *Genome Biol* **19**, 78, doi:10.1186/s13059-018-1449-6 (2018).

12 Kim, D. *et al.* TopHat2: accurate alignment of transcriptomes in the presence of insertions, deletions and gene fusions. *Genome Biol* **14**, R36, doi:10.1186/gb-2013-14-4-r36 (2013).

13 Butler, A., Hoffman, P., Smibert, P., Papalexi, E. & Satija, R. Integrating single-cell transcriptomic data across different conditions, technologies, and species. *Nat Biotechnol* **36**, 411-420, doi:10.1038/nbt.4096 (2018).

14 Langmead, B. & Salzberg, S. L. Fast gapped-read alignment with Bowtie 2. *Nat Methods* **9**, 357-359, doi:10.1038/nmeth.1923 (2012).

15 Anders, S., Pyl, P. T. & Huber, W. HTSeq--a Python framework to work with high-throughput sequencing data. *Bioinformatics* **31**, 166-169, doi:10.1093/bioinformatics/btu638 (2015).

16 Love, M. I., Huber, W. & Anders, S. Moderated estimation of fold change and dispersion for RNA-seq data with DESeq2. *Genome Biol* **15**, 550, doi:10.1186/s13059-014-0550-8 (2014).
